# Subsequent Hypertension’s Mediation of the Association Between Sleep Duration Trajectories and New-Onset Cardiovascular Disease: Population-Based Cohort Study

**DOI:** 10.2196/78914

**Published:** 2026-05-28

**Authors:** Hongwei Liu, Minghui Wu, Minheng Zhang, Jian Pan, Haiyan Wu, Haixia Fan

**Affiliations:** 1 Department of Neurology, Taiyuan City Central Hospital, taiyuan, China; 2 Department of Gerontology, The First People's Hospital of Jinzhong, Jinzhong, China; 3 Department of Anesthesiology, Shenzhen People's Hospital Longhua Branch, ShenZhen, China; 4 Department of Sleep Center, First Hospital of Shanxi Medical University, 85, Jiefang South Road, Taiyuan, 030001, China, 86 13623515703

**Keywords:** sleep duration trajectory, hypertension, cardiovascular disease, stroke, heart disease, longitudinal study, mediation analysis

## Abstract

**Background:**

Prior research has rarely explored the link between sleep duration trajectories and cardiovascular disease (CVD) either in cross-sectional or longitudinal data.

**Objective:**

We aimed to analyze the relationship between sleep duration trajectories and the incidence of CVD.

**Methods:**

Data from 5603 participants in the China Health and Retirement Longitudinal Study (CHARLS) were used in the analysis. Sleep duration was self-reported at multiple time points, and group-based trajectory modeling identified distinct patterns of total and nocturnal sleep duration over time. To investigate the link among sleep duration trajectories and CVD, Cox proportional hazards models, restricted cubic splines, and mediation analyses were used, with sensitivity analyses ensuring the results’ robustness.

**Results:**

Four sleep duration trajectories were identified, including steady high, inverted U-shaped, steady low, and U-shaped. In fully adjusted models, an inverted U-shaped sleep trajectory was associated with a 34% higher risk of hypertension compared with the steady high group (total sleep: hazard ratio [HR]=1.34, 95% CI 1.07‐1.69; *P*=.01). Notably, the inverted U-shaped sleep trajectory showed a 110% increased stroke risk during nocturnal periods compared to the steady high group (nocturnal sleep: HR=2.10, 95% CI 1.08‐4.05; *P*=.03). The findings remained consistent across a range of sensitivity analyses. Hypertension was identified as a partial mediator of the association between sleep instability and stroke risk.

**Conclusions:**

The findings indicate that an inverted U-shaped sleep pattern is associated with increased cardiovascular risk, partially mediated by hypertension, highlighting sleep stability as a potential preventive target.

## Introduction

Due to longer life expectancy and declining fertility rates, the global population aged 60 years and older is expected to rise from 12% to 22% between 2015 and 2050 [1]. China, one of the fastest-aging countries, will see the percentage of people aged >60 years grow from 12.4% in 2010 to 28% by 2040, reaching around 397 million [2]. Cardiovascular diseases (CVD) [3], including hypertension [4], stroke [5], and heart diseases [6], are particularly significant, as they represent leading causes of morbidity and mortality in older adults [7]. As the aging population increases, the burden of CVD will intensify, placing additional pressure on health care systems globally. In light of this, strategies to improve cardiovascular health and promote healthy aging are essential to reduce the impact of these diseases and enhance the quality of life for older adult individuals.

Insufficient or excessive sleep has been linked to adverse outcomes [8], including mood disorders [9], cognitive frailty [10], cancers [11], and even mortality [12]. Recent studies have shown that sleep abnormalities, including both deprivation and excessive sleep, negatively impact physiological functions and notably increase the risk of CVDs. Both short and long sleep durations are strongly linked to an elevated risk of coronary heart disease [13] and stroke [14]. Short sleep duration is strongly associated with a higher incidence of hypertension, while the link between long sleep duration or difficulty falling asleep and hypertension remains inconclusive [15]. Although these studies explored the link between sleep duration and CVD, their conclusions were mixed. Most research relies on cross-sectional surveys and neglects the continuous development of sleep duration, offering an incomplete view of how it changes throughout life. This gap is particularly pertinent in middle-aged and older adults, where sleep patterns often change due to physiological, psychological, and social factors. Tracking these changes over time, rather than using a single measurement, is crucial for understanding their role in CVD development.

In light of these concerns, this study seeks to investigate the association between sleep duration trajectories and CVD outcomes in Chinese middle-aged and older adults (the China Health and Retirement Longitudinal Study [CHARLS]). By using a longitudinal dataset, we aim to identify distinct sleep trajectories over time and examine their potential role in the onset of CVD. This research not only contributes to the growing body of literature on sleep and health outcomes but also has important implications for public health strategies aimed at improving the quality of life and reducing the burden of CVD among the aging population.

## Methods

### Study Design and Participants

**Figure 1.**
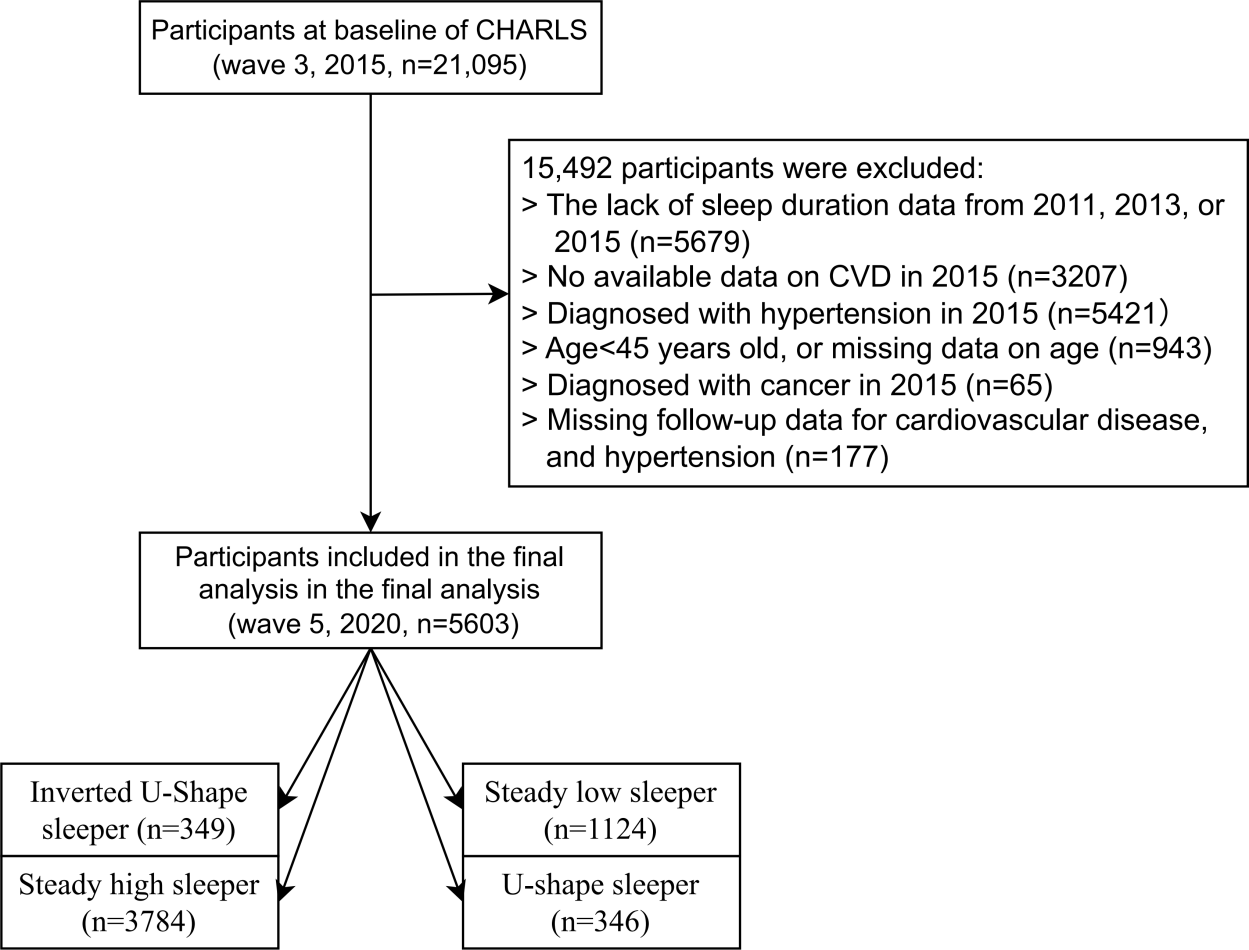
Flowchart of participants’ selection. CVD: cardiovascular disease; CHARLS: China Health and Retirement Longitudinal Study.

This nationally representative longitudinal survey, CHARLS, is designed to explore the health and economic status of Chinese people aged 45 years and older [16]. The CHARLS study’s design, methods, and participant information have been extensively described in prior publications [16]. The 2011 baseline survey (Wave 1) involved 17,708 participants from over 10,000 households, with follow-up surveys conducted in 2013 (Wave 2), 2015 (Wave 3), 2018 (Wave 4), and 2020 (Wave 5). The research adhered to the STROBE (Strengthening the Reporting of Observational Studies in Epidemiology) standards for reporting observational epidemiological studies. Data from Waves 1, 2, and 3 (2011, 2013, and 2015) were used in this study to track sleep duration trajectories. The 2015 Wave 3 of the CHARLS initially assessed 21,095 participants. Participants were excluded sequentially for the following reasons: missing sleep duration data in 2011, 2013, or 2015 (n=5679); lack of CVD data in 2015 (n=3207); preexisting hypertension in 2015 (n=5421); age <45 years or missing age information (n=943); diagnosis of cancer in 2015 (n=65); and missing follow-up information on CVD or hypertension (n=177). With these exclusions, 5603 participants were included in the final longitudinal analysis, which was followed up through Wave 5 (2020) (Figure 1).

### Measurements of Sleep Duration

Sleep duration trajectories refer to patterns of change in an individual’s total sleep duration, which includes both nighttime sleep and daytime naps, over a specific period [17]. By using longitudinal trajectories, we were able to capture long-term sleep behavior patterns, including the consistency and changes in sleep duration over multiple years, providing a more detailed characterization of sleep patterns than any single metric. Detailed data on nighttime sleep and daytime naps were obtained from the 2011, 2013, and 2015 CHARLS waves, and the total sleep duration over 24 hours was calculated for each participant at each wave. The selection of these 3 waves was intentional, as they cover all sleep assessments available before the 2015 landmark, ensuring complete observation of exposure information before follow-up starts. Moreover, latent class and longitudinal trajectory models require at least 3 repeated measurements to reliably distinguish stable, monotonic, and nonlinear patterns, meaning that using only the 2011 and 2013 waves would not provide sufficient temporal variation for meaningful trajectory identification. Surveys conducted in 2018 or 2020, which are part of the post-2015 waves, were excluded due to methodological considerations. Data on sleep duration collected after 2015 may have been influenced by intervening hypertension or cardiovascular events, leading to misclassification of exposure after the outcome and violating the required temporal order for valid conclusions. By extending the trajectory window to 2018, substantial survivor bias would be introduced, as only those who remained event-free and continued follow-up until 2018 could contribute full sleep data, resulting in a healthier, nonrepresentative sample. Focusing trajectory construction on the pre–follow-up period (2011‐2015) offers the temporal resolution required to capture significant sleep dynamics while upholding a valid exposure-outcome framework for subsequent cardiovascular evaluations. Applying latent class mixed models (LCMM), we identified 4 distinct sleep duration trajectory groups, including inverted U-shaped sleeper, steady low sleeper, steady high sleeper, and U-shaped sleeper. Each group represents a unique sleep pattern and trend. Inverted U-shaped sleepers show an initial increase in sleep duration, followed by a peak and a gradual decrease, potentially reflecting changes in sleep patterns during different physiological or life stages in middle-aged and older adults. Steady low sleepers maintain consistently low sleep durations with minimal fluctuation, which may indicate chronic sleep deprivation, potentially linked to cognitive decline, mental health issues, and other health concerns. Steady high sleepers consistently maintain higher sleep durations, which could reflect healthy sleep habits or specific health needs, such as restorative sleep, although prolonged high sleep duration may also be associated with chronic diseases or health risks, including metabolic disorders. Finally, U-shaped sleepers exhibit a decrease in sleep duration followed by an increase, forming a U-shaped trajectory, which may reflect dynamic changes in sleep needs across different age groups or health conditions, highlighting physiological, psychological, and environmental adaptations during the aging process.

### Outcome Identification

Data on cardiovascular outcomes came from self-reported physician diagnoses collected in Waves 4 (2018) and 5 (2020). In accordance with established epidemiological practice, CVD was defined strictly as the first occurrence of stroke or heart disease, and hypertension was treated as a separate end point rather than included within the CVD composite [18-20]. Participants were classified as having incident CVD if they answered “yes” to either of the following physician-diagnosis questions, “Have you ever been diagnosed by a doctor with a heart attack, angina, coronary heart disease, heart failure, or other cardiovascular problems?” or “Have you received a diagnosis of a stroke from a doctor?” [18-20]. The event date was defined as the first survey wave (2018 or 2020) at which the condition was reported. If the same condition was reported in later waves, these were treated as duplicate events, and only the earliest report was used for analysis. When concurrent events (stroke and heart disease reported in the same wave) occurred, the participant was counted as having experienced a CVD event, with the shared date assigned as the event time. Each individual could contribute a maximum of one event to the hypertension end point and one event to the cardiovascular end points. In defining CVD as a combination of stroke and heart disease, only the first diagnosis of either was acknowledged as the incident CVD event, with subsequent reports considered duplicates.

In Waves 4 (2018) and 5 (2020), data regarding hypertension were obtained through a standardized self-reported physician-diagnosis questionnaire. Instead of incorporating it into the CVD composite, hypertension was addressed as an independent end point. Participants were classified as having incident hypertension if they answered “yes” to the question, “Have you been told by a doctor that you have high blood pressure?” The initial survey wave (2018 or 2020) that recorded a physician’s diagnosis of hypertension was used to define the event date. Subsequent reports were regarded as duplicates, and only the earliest one was included in the analysis. Each participant was limited to contributing one hypertension event, irrespective of their classification for stroke, heart disease, or composite CVD.

### Covariates

Covariates were selected based on established literature and clinical expertise. These covariates included age, gender, marital status, education level, smoking status, alcohol consumption, hypertension, dyslipidemia, heart disease, obesity, systolic blood pressure (SBP), diastolic blood pressure (DBP), heart rate, waist circumference, waist-to-height ratio, high-density lipoprotein cholesterol, total cholesterol, hemoglobin A_1c_ (HbA_1c_), triglycerides, low-density lipoprotein cholesterol, creatinine, blood urea nitrogen (BUN), fasting plasma glucose (FPG), BMI, and estimated glomerular filtration rate (eGFR). Hypertension was defined as a self-reported physician diagnosis, use of antihypertensive medications, and/or an average SBP of ≥140 mm Hg and/or DBP of ≥90 mm Hg [21]. Diabetes mellitus (DM) was defined as FPG of ≥126 mg/dL or HbA_1c_ level of ≥6.5%, and/or a self-reported physician diagnosis, and/or the use of glucose-lowering medications. Pre-DM was identified by FPG levels of 100‐125 mg/dL or HbA_1c_ level between 5.7% and 6.4%. Individuals without DM or pre-DM were classified as having normal glucose regulation [22]. Dyslipidemia was diagnosed based on a self-reported physician diagnosis, the use of lipid-lowering medications, and/or triglycerides >150 mg/dL, high-density lipoprotein cholesterol level of <40 mg/dL, low-density lipoprotein cholesterol level of >160 mg/dL, or total cholesterol level of >240 mg/dL [23]. Chronic kidney disease was defined as a self-reported physician diagnosis or an eGFR of <60 mL/min/1.73 m², in accordance with previous CHARLS studies [24]. Obesity was assessed by calculating BMI using the formula: weight (kg) / height² (m²) [25]. Consistent with our earlier research [26,27], socioeconomic status (SES) was assessed through 4 indicators, including yearly household income, educational achievement, employment status, and medical insurance coverage. Family annual income was determined by the total household income from the prior year and divided into 2 groups, above and below the median, with the sample median set at US $2877.7 or CNY 18,130.00. Education was sorted into 3 tiers, including below high school, high school or equivalent (General Educational Development), and college or more advanced. Educational attainment was classified into 3 categories, including junior high school or below, senior high school, and college or above. Employment status was sorted into employed (covering those with paid employment, self-employment, or retired) and unemployed. Insurance coverage was divided into 2 categories, insured, which includes public or private health insurance, and uninsured.

### Statistical Analyses

For continuous variables that did not conform to a normal distribution, medians and IQRs were used, and categorical variables were displayed as frequencies and percentages. The Kruskal-Wallis rank-sum test was used to compare nonnormally distributed continuous variables across the 4 groups, and the chi-square test was used for categorical variables. With the assumption that the missing data followed a random pattern, we used *multiple imputation by chained equations* (MICE) in R (R Foundation for Statistical Computing) to estimate the missing covariates. Incorporating all analytic covariates and auxiliary variables, the imputation model aimed to maximize the use of information. To achieve algorithmic stability and convergence, 5 complete datasets were generated, each undergoing 50 iterations. Predictive mean matching (“pmm”) was used for continuous variables, logistic regression (“logreg”) for binary variables, multinomial models for nominal categorical variables, and proportional-odds models for ordinal variables. The evaluation of convergence involved inspecting trace plots for the mean and SD of the imputed values. Each imputed dataset was analyzed with Cox proportional hazards models, and the parameter estimates were merged using Rubin rules to generate pooled coefficients and standard errors. Using the *LCMM* package in R, we applied LCMMs to study longitudinal variations in total and nighttime sleep duration. Models with different polynomial specifications (linear, quadratic, and cubic) were compared to evaluate goodness of fit, and the optimal model was determined according to the principle of Bayesian Information Criterion minimization, ensuring both statistical adequacy and practical interpretability (Table S1 and S2 in Multimedia Appendix 1). To maintain enough sample representation in each trajectory and prevent overfitting, each latent class needed to account for at least 5% of the total sample. According to these guidelines, a 4-class model provided the optimal fit, resulting in 4 distinct sleep trajectory patterns, including inverted U-shaped sleepers, steady low sleepers, steady high sleepers, and U-shaped sleepers. Using Schoenfeld residuals, the proportional hazards assumption in the Cox models was evaluated, and no significant violations were identified (all *P*>.05). Variance inflation factors were used to evaluate potential multicollinearity among covariates, and since all values were below 5, it indicated no serious collinearity. These diagnostic checks validated that the Cox proportional hazards models complied with crucial assumptions and were apt for the analyses. Potential confounders were pinpointed using prior epidemiological evidence and clinical relevance, encompassing demographic features, lifestyle practices, and metabolic indicators. Using Cox proportional hazards models, we investigated the relationship between sleep patterns and hypertension, as well as subsequent cardiovascular events, including stroke and heart disease. To elucidate the progressive impact of confounding, 3 hierarchical Cox proportional hazards models were fitted. Model 1 served as the crude model; Model 2 adjusted for sociodemographic and lifestyle variables (age, sex, education level, marital status, smoking, and alcohol use); and Model 3 extended the adjustment to biological variables, namely dyslipidemia, BUN, eGFR, DM, and obesity status, thereby capturing potential physiological confounding. Follow-up time from Wave 4 (2018) to Wave 5 (2020) or event occurrence served as the analytic time scale. Participants who remained free of cardiovascular events were administratively censored at their last observed follow-up. Restricted cubic spline models were applied to examine the potential nonlinear associations between sleep duration and cardiovascular outcomes. Based on established recommendations, 4 knots were set at the 5th, 35th, 65th, and 95th percentiles of the sleep-duration distribution to fit restricted cubic spline models. To enhance clinical interpretability, contrasts were determined at sleep durations of 6, 7, 9, and 10 hours. These values were selected based on both clinical relevance and the empirical distribution observed in the trajectory patterns. These values were selected a priori because they correspond to commonly used sleep duration categories in epidemiological research, with approximately 6 hours representing short sleep, 7‐8 hours reflecting recommended sleep duration, and 9‐10 hours representing extended sleep. Using these established thresholds allows the spline-estimated associations to be interpreted against clinically meaningful reference points without imposing additional assumptions on the functional form. An exploratory mediation analysis was conducted to see if hypertension could potentially serve as an intermediate pathway between total or nocturnal sleep trajectories and cardiovascular outcomes. Using nonparametric bootstrapping, direct and indirect effects were estimated, but the temporal structure of the data hinders causal interpretation, indicating that the findings are exploratory. For this analysis, the *mediation* package was used with 1000 iterations of simulation. To mitigate potential reverse causality and enhance the robustness of our findings, participants who experienced cardiovascular events within the first 2 years of follow-up were excluded from the analysis. In addition, to account for uncertainty in sleep trajectory assignment, sensitivity analyses used misclassification-adjusted Cox regression models with posterior-probability weighting. Further sensitivity analyses included additional adjustments for socioeconomic status to evaluate the possible influence of leftover socioeconomic confounding. R software (version 4.4.1) facilitated all statistical analyses, with a 2-sided *P* value less than .05 considered statistically significant.

### Ethical Considerations

The Biomedical Ethics Review Committee of Peking University (IRB00001052-11015) approved the study, and all participants provided written informed consent. All data used in this study were obtained from publicly available, deidentified datasets. These datasets were generated by original investigators who obtained informed consent from participants and conducted their studies in accordance with the Declaration of Helsinki and applicable institutional review board approvals. No new human participants were enrolled for this research, and no additional ethical approval was required for this secondary analysis. All data accessed are publicly available and fully anonymized prior to deposition. No personally identifiable information (such as names, addresses, contact details, or unique personal identifiers) was used or disclosed in any part of the analysis. We strictly adhered to the terms of use and data sharing policies of the original repositories. All analyses were conducted on aggregated or deidentified data, ensuring that individual privacy and confidentiality were fully maintained throughout the study. As this research involved the analysis of preexisting, deidentified data, no participants were directly involved in study procedures and no compensation was provided or required as part of this work.

## Results

### Baseline Characteristics of Participants and Sleep Duration Trajectories

**Figure 2.**
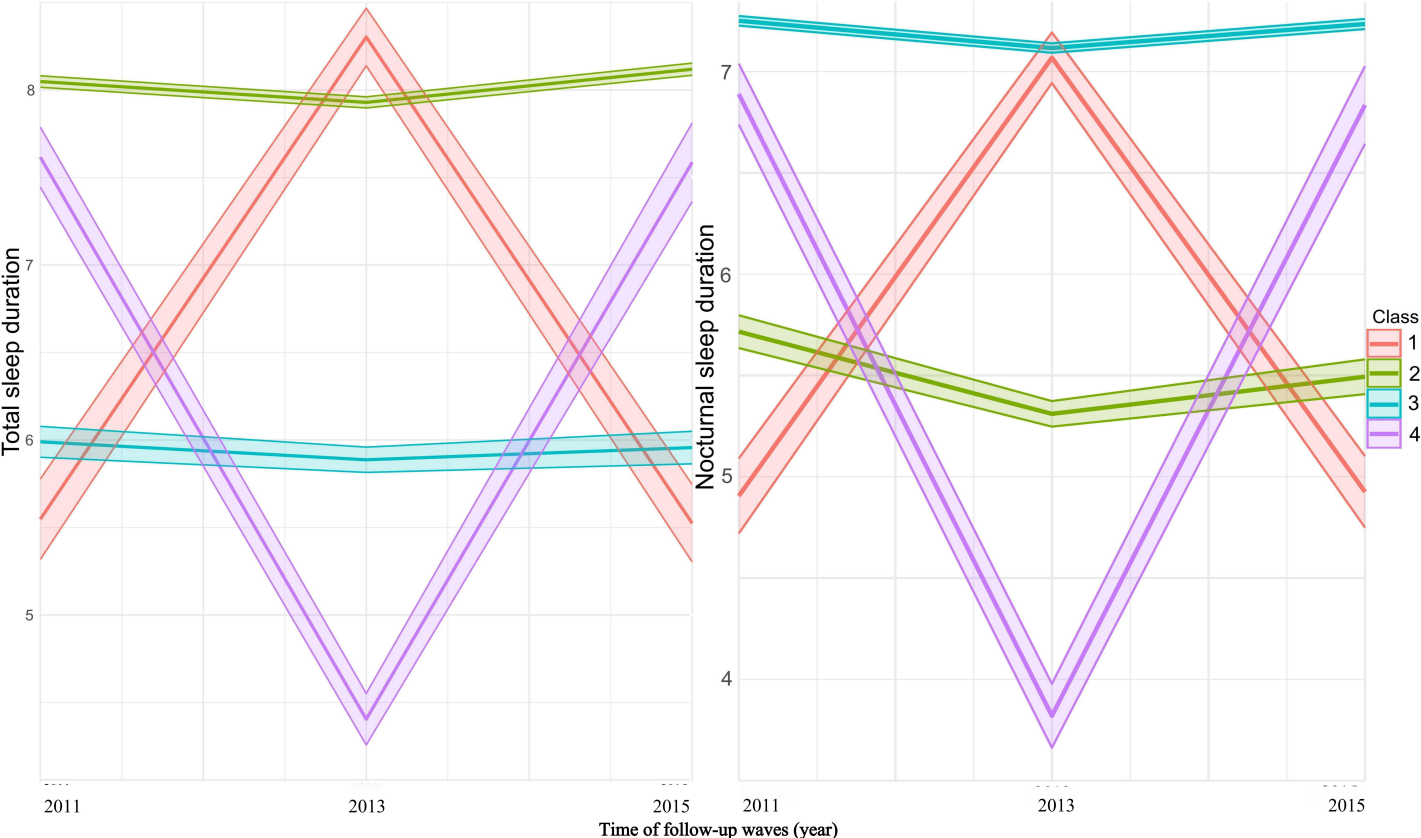
Total sleep duration trajectories from 2011 to 2015 in Chinese middle-aged and older adults. (**A**) Total sleep duration trajectories. (**B**) Nocturnal sleep duration trajectories.

Table 1 summarized the baseline demographic and clinical characteristics of 5603 participants, grouped by total sleep duration trajectories, including inverted U-shaped (n=349), steady low (n=1124), steady high (n=3784), and U-shaped (n=346). Figures 2A and 2B further illustrated the longitudinal trends in total and nocturnal sleep duration from 2011 to 2015, confirming 4 distinct trajectories. The overall median age was 60 (IQR 53.00‐66.00) years, with group-specific medians ranging from 62 (IQR 52.00‐68.00) years in the inverted U-shaped group to 61 (IQR 53.00‐68.00) years in the U-shaped group. The cohort included 2765 (47.74%) males and 2928 (52.26%) females, with the proportion of females highest in the U-shaped group (65.90%).

**Table 1.** Baseline characteristics of participants categorized by total sleep duration trajectories.

Characteristic	Overall (N=5603)	Inverted U-shape sleeper (n=349)	Steady high sleeper (n=3784)	Steady low sleeper (n=1124)	U-shaped sleeper (n=346)	*P* value^a^
Sleep (total), median (IQR)	7.00 (6.00-8.33)	4.50 (3.00-6.00)	8.00 (7.00-9.00)	5.00 (4.00-6.00)	7.00 (5.50-9.00)	<.001
Sleep (night), median (IQR)	6.50 (5.00-8.00)	4.00 (3.00-6.00)	7.00 (6.00-8.00)	5.00 (4.00-6.00)	7.00 (5.00-8.00)	<.001
Age (years), median (IQR)	60.00 (53.00-66.00)	62.00 (56.00-68.00)	59.00 (53.00-65.00)	61.00 (54.00-67.00)	61.00 (53.00-68.00)	<.001
Sex, n (%)						<.001
Male	2675.00 (47.74)	136.00 (38.97)	1963.00 (51.88)	458.00 (40.75)	118.00 (34.10)	
Female	2928.00 (52.26)	213.00 (61.03)	1,821.00 (48.12)	666.00 (59.25)	228.00 (65.90)	
Marital status, n (%)						<.001
Married and living with spouse	4977.00 (88.83)	304.00 (87.11)	3,418.00 (90.33)	958.00 (85.23)	297.00 (85.84)	
Other	626.00 (11.17)	45.00 (12.89)	366.00 (9.67)	166.00 (14.77)	49.00 (14.16)	
Education level, n (%)						.008
Junior high school and below	886.00 (15.81)	61.00 (17.48)	561.00 (14.83)	195.00 (17.35)	69.00 (19.94)	
Senior high school	326.00 (5.82)	13.00 (3.72)	240.00 (6.34)	62.00 (5.52)	11.00 (3.18)	
College and above	4,391.00 (78.37)	275.00 (78.80)	2,983.00 (78.83)	867.00 (77.14)	266.00 (76.88)	
Smoking status, n (%)						<.001
Never smoked	3,298.00 (58.86)	233.00 (66.76)	2120.00 (56.03)	709.00 (63.08)	236.00 (68.21)	
Smoking at the time of this writing	1659.00 (29.61)	78.00 (22.35)	1209.00 (31.95)	291.00 (25.89)	81.00 (23.41)	
Ever smoked	646.00 (11.53)	38.00 (10.89)	455.00 (12.02)	124.00 (11.03)	29.00 (8.38)	
Drinking status, n (%)						.005
Never drink	3774.00 (67.36)	245.00 (70.20)	2506.00 (66.23)	777.00 (69.13)	246.00 (71.10)	
Drinking at the time of this writing	1493.00 (26.65)	73.00 (20.92)	1063.00 (28.09)	277.00 (24.64)	80.00 (23.12)	
Ever drunk	336.00 (6.00)	31.00 (8.88)	215.00 (5.68)	70.00 (6.23)	20.00 (5.78)	
DM^b^, n (%)						.05
No	3558.00 (63.50)	227.00 (65.04)	2378.00 (62.84)	747.00 (66.46)	206.00 (59.54)	
Yes	2045.00 (36.50)	122.00 (34.96)	1406.00 (37.16)	377.00 (33.54)	140.00 (40.46)	
Dyslipidemia, n (%)						.17
No	4044.00 (72.18)	260.00 (74.50)	2703.00 (71.43)	836.00 (74.38)	245.00 (70.81)	
Yes	1559.00 (27.82)	89.00 (25.50)	1081.00 (28.57)	288.00 (25.62)	101.00 (29.19)	
Obesity, n (%)						<.001
Normal weight	3119.00 (55.67)	229.00 (65.62)	2030.00 (53.65)	660.00 (58.72)	200.00 (57.80)	
Overweight	1833.00 (32.71)	94.00 (26.93)	1293.00 (34.17)	343.00 (30.52)	103.00 (29.77)	
Obesity	651.00 (11.62)	26.00 (7.45)	461.00 (12.18)	121.00 (10.77)	43.00 (12.43)	
Creatinine (mg/dl), median (IQR)	0.85 (0.70-1.79)	0.84 (0.68-1.23)	0.86 (0.70-1.93)	0.84 (0.69-1.32)	0.85 (0.67-1.79)	<.001
BUN^c^ (mg/dl), median (IQR)	15.69 (12.89-19.89)	15.41 (12.61-19.89)	15.69 (13.17-20.17)	15.69 (12.89-19.33)	15.41 (12.61-20.17)	.04
eGFR^d^ (ml/min/1.73m2), median (IQR)	86.80 (38.43-98.00)	86.51 (57.05-96.28)	87.27 (33.43-98.43)	86.48 (55.74-97.24)	82.86 (33.89-97.63)	.26
Hypertension incident, n (%)						.11
No	5165.00 (92.18)	313.00 (89.68)	3508.00 (92.71)	1025.00 (91.19)	319.00 (92.20)	
Yes	438.00 (7.82)	36.00 (10.32)	276.00 (7.29)	99.00 (8.81)	27.00 (7.80)	
Stroke incident, n (%)						.02
No	5503.00 (98.22)	340.00 (97.42)	3731.00 (98.60)	1096.00 (97.51)	336.00 (97.11)	
Yes	100.00 (1.78)	9.00 (2.58)	53.00 (1.40)	28.00 (2.49)	10.00 (2.89)	
Heart incidence, n (%)						.11
No	5165.00 (92.18)	313.00 (89.68)	3508.00 (92.71)	1025.00 (91.19)	319.00 (92.20)	
Yes	438.00 (7.82)	36.00 (10.32)	276.00 (7.29)	99.00 (8.81)	27.00 (7.80)	
CVD^e^ incident, n (%)						.006
No	5081.00 (90.68)	304.00 (87.11)	3465.00 (91.57)	1002.00 (89.15)	310.00 (89.60)	
Yes	522.00 (9.32)	45.00 (12.89)	319.00 (8.43)	122.00 (10.85)	36.00 (10.40)	

a Kruskal-Wallis rank sum test; Pearson *χ*2 test; Data were presented as mean (SD), median (IQR), or as n (%).

b DM: diabetes mellitus.

c BUN: blood urea nitrogen.

d eGFR: estimated glomerular filtration rate.

e CVD: cardiovascular disease.

Both total sleep duration and nocturnal sleep duration differed significantly across sleep trajectory groups (*P*<.001). Participants in the inverted U-shaped sleep group exhibited the shortest median total sleep duration (4.5, IQR 3.0‐6.0 h) and nocturnal sleep duration (4.0, IQR 3.0‐6.0 h), whereas those in the steady high sleep group showed the longest median total sleep duration (8.0, IQR 7.0‐9.0 h) and nocturnal sleep duration (7.0, IQR 6.0‐8.0 h). Participants differed significantly across trajectory groups regarding age, gender, marital status, education level, smoking status, drinking status, creatinine, BUN, and obesity (all *P*<.05; Table 1). Although no significant differences were observed in estimated glomerular filtration rate (*P*=.26), DM (*P*=.05), dyslipidemia (*P*=.17), or hypertension (*P*=.11; Table 1).

### Association of Sleep Duration Trajectories With New-Onset Hypertension

**Figure 3.**
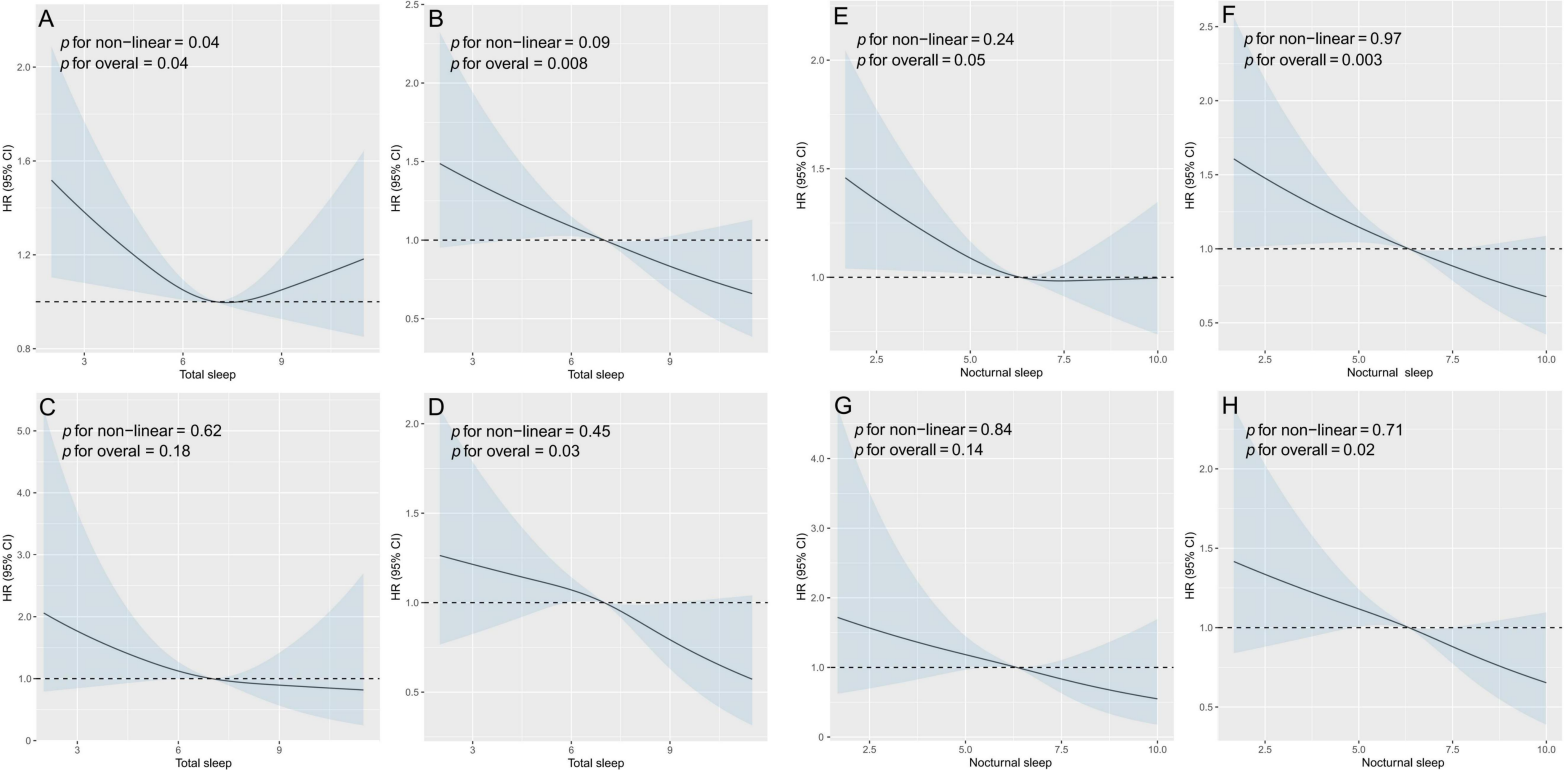
Curvilinear association between total sleep/nocturnal sleep and risk of cardiovascular outcomes. (A, E) New onset of hypertension; (B, F) new onset of cardiovascular diseases (CVD); (C, G) new onset of stroke; and (D, H) new onset of heart diseases. HR: hazard ratio; CVD: cardiovascular disease.

In the course of the follow-up, 1123 new hypertension cases were identified, making up 20% (Table 2). Differences were observed in the onset of hypertension among the sleep trajectory groups. The distribution of total sleep trajectories (Figure S1A in Multimedia Appendix 2) was 19% for steady high sleepers, 22% for steady low sleepers, 24% for inverted U-shaped sleepers, and 20% for U-shaped sleepers. Nocturnal sleep trajectories (Figure S1B in Multimedia Appendix 2) exhibited a similar pattern, with incidences of 24%, 21%, 19%, and 22% across the respective categories. For total sleep duration trajectories, compared with the steady high group (reference), participants in the inverted U-shaped group had a significantly higher risk of developing hypertension (hazard ratio [HR]=1.34, 95% CI 1.07‐1.69; *P*=.01) in the fully adjusted model (Model 3; Table 2). The steady low and U-shaped groups showed modest but nonsignificant increases in hypertension risk HR=1.13, 95% CI 0.97‐1.31; *P*=.11; and HR=0.98, 95% CI 0.76‐1.26; *P*=.87, respectively; Table 2). Figure 3A illustrates the association between total sleep duration and the risk of incident hypertension. The overall association was statistically significant (for overall *P*=.04), and the test for nonlinearity also reached significance (for nonlinear *P*=.04).

**Table 2.** Association of the sleep trajectories and new-onset hypertension.

Characteristic	N	Event, n (%)	Model 1^a^		Model 2^b^		Model 3^c^	
			HR^d^ (95% CI)	*P* value	HR (95% CI)	*P* value	HR (95% CI)	*P* value
Total sleep trajectories	5603	1123 (20.04)						
Steady high sleeper	3784	726 (19)	Reference^e^		Reference		Reference	
Steady low sleeper	1124	242 (22)	1.14 (0.98-1.31)	.09	1.11 (0.96-1.29)	.17	1.13 (0.97-1.31)	.11
Inverted U-shaped sleeper	349	85 (24)	1.31 (1.05-1.64)	.02	1.26 (1.01-1.59)	.04	1.34 (1.07-1.69)	.01
U-shaped sleeper	346	70 (20)	1.05 (0.82-1.34)	.69	1.03 (0.80-1.31)	.84	0.98 (0.76-1.26)	.87
Nocturnal sleep trajectories	5603	1123 (20.04)						
Steady high sleeper	3849	744 (19)	Reference		Reference		Reference	
Steady low sleeper	1073	224 (21)	1.09 (0.94-1.26)	.26	1.07 (0.92-1.24)	.41	1.06 (0.91-1.24)	.43
Inverted U-shaped sleeper	386	91 (24)	1.24 (1.00-1.54)	.05	1.19 (0.96-1.48)	.12	1.26 (1.01-1.58)	.04
U-shaped sleeper	295	64 (22)	1.13 (0.87-1.45)	.37	1.10 (0.85-1.42)	.48	1.09 (0.84-1.41)	.50

a Model 1: crude model.

b Model 2: adjusted for age, gender, education level, marital status, drinking status, and smoking status.

c Model 3: adjusted for age, gender, education level, marital status, drinking status, smoking status, dyslipidemia (current use of lipid-lowering medications, and/or triglycerides>150 mg/dL, high-density lipoprotein cholesterol [HDL-C]<40 mg/dL, low-density lipoprotein cholesterol [LDL-C]>160 mg/dL, or total cholesterol [TC]>240 mg/dL), blood urea nitrogen, estimated glomerular filtration rate, diabetes mellitus, and obesity status.

d HR: hazard ratio.

e Participants with steady high sleep duration, which is used as the reference for comparison when reporting hazard ratios (HR) for the other sleep trajectory groups.

For nocturnal sleep duration trajectories, similar patterns were observed. The inverted U-shaped group had a significantly elevated risk of new-onset hypertension compared to the steady-high group (HR=1.26, 95% CI 1.01‐1.58; *P*=.04 in Model 3). No significant associations were found for the steady low (HR=1.13, 95% CI 0.97‐1.30; *P*=.11) or U-shaped group (HR=0.97, 95% CI 0.74‐1.26; *P*=.81). Figure 3E depicts the association between nocturnal sleep duration and hypertension risk. The overall association was statistically significant (*P* for overall=.05), whereas the test for nonlinearity was not significant (*P* for nonlinear=.24).

### Association of Sleep Duration Trajectories With New-Onset CVD

Over the follow-up duration, 522 participants, which is 10.17%, developed new-onset CVD. Different sleep trajectory groups also experienced varying rates of new CVD onset. The sleep trajectory analysis (Figure S1C in Multimedia Appendix 2) showed that 8.4% of participants were classified as steady high sleepers, 11.0% as steady low sleepers, 13.0% as inverted U-shaped sleepers, and 10.0% as U-shaped sleepers. For nocturnal sleep trajectories (Figure S1D in Multimedia Appendix 2), the corresponding rates were 12%, 11%, 8.5%, and 11%, respectively. Compared to steady high sleepers (reference), those categorized as inverted U-shaped showed an increased chance of experiencing new-onset CVD. Compared to steady high sleepers, the inverted U-shaped group showed a nearly statistically significant elevated risk in the fully adjusted model, with an HR of 1.46 (95% CI 1.07‐2.00; *P*=.02) in Model 3 (Table 3). Higher cardiovascular risk was observed in the U-shaped and steady low groups, but the associations were not statistically significant (HR=1.08 95% CI 0.76‐1.54; *P*=.66; HR=1.22, 95% CI 0.99‐1.50; *P*=.07; Table 3). In Figure 3B, the correlation between total sleep duration and the occurrence of new CVD is shown. The overall association was statistically significant (*P* for overall=.008), while the test for nonlinearity did not indicate evidence of a nonlinear relationship (*P* for nonlinearity=.09).

**Table 3.** Association of the sleep trajectories and new-onset cardiovascular disease.

Characteristic	N	Event, n (%)	Model 1^a^		Model 2^b^		Model 3^c^	
			HR^d^ (95% CI)	*P* value	HR (95% CI)	*P* value	HR (95% CI)	*P* value
Total sleep trajectories	5603	522 (9.32)						
Steady high sleeper	3784	319 (8.4)	Reference^e^		Reference		Reference	
Steady low sleeper	1124	122 (11)	1.31 (1.06-1.61)	.01	1.21 (0.98-1.49)	.08	1.22 (0.99-1.50)	.07
Inverted U-shaped sleeper	349	45 (13)	1.58 (1.15-2.15)	.004	1.40 (1.02-1.92)	.04	1.46 (1.07-2.00)	.02
U-shaped sleeper	346	36 (10)	1.25 (0.88-1.76)	.21	1.11 (0.79-1.57)	.55	1.08 (0.76-1.54)	.65
Nocturnal sleep trajectories	5603	522 (9.32)						
Steady high sleeper	3849	329 (8.5)	Reference		Reference		Reference	
Steady low sleeper	1073	117 (11)	1.30 (1.05-1.60)	.02	1.20 (0.97-1.48)	.10	1.21 (0.98-1.50)	.08
Inverted U-shaped sleeper	386	45 (12)	1.40 (1.02-1.91)	.04	1.24 (0.90-1.70)	.18	1.30 (0.95-1.79)	.10
U-shaped sleeper	295	31 (11)	1.24 (0.85-1.79)	.26	1.11 (0.76-1.60)	.59	1.11 (0.77-1.61)	.57

a Model 1: crude model.

b Model 2: adjusted for age, gender, education level, marital status, drinking status, and smoking status.

c Model 3: adjusted for age, gender, education level, marital status, drinking status, smoking status, dyslipidemia (current use of lipid-lowering medications, and/or triglycerides>150 mg/dL, high-density lipoprotein cholesterol [HDL-C]<40 mg/dL, low-density lipoprotein cholesterol [LDL-C]>160 mg/dL, or total cholesterol [TC]>240 mg/dL), blood urea nitrogen, estimated glomerular filtration rate, diabetes mellitus, and obesity status.

d HR: hazard ratio.

e Participants with steady high sleep duration, which is used as the reference for comparison when reporting hazard ratios (HR) for the other sleep trajectory groups.

When evaluating nocturnal sleep duration trajectories, the overall association patterns remained consistent. Compared with steady high sleepers (reference), the steady low sleep trajectory was associated with an elevated risk of incident CVD in the fully adjusted model, although the association did not reach statistical significance (HR=1.21, 95% CI 0.98‐1.50; *P*=.08; Table 3). Similarly, individuals with an inverted U-shaped sleep trajectory showed a higher risk of new-onset CVD, but this association was marginal and not statistically significant (HR=1.30, 95% CI 0.95‐1.79; *P*=.10). No significant association was observed for the U-shaped sleep trajectory (HR=1.11, 95% CI 0.77‐1.61; *P*=.57; Table 3). Figure 3F depicts the association between nocturnal sleep duration and heart disease risk. The nonlinearity test did not indicate evidence of a nonlinear relationship (*P* for nonlinear=.96), whereas the overall association was highly significant (*P* for overall=.003), mirroring the pattern observed for total sleep duration.

### Association of Sleep Duration Trajectories With New-Onset Stroke

In the course of the study, 100 individuals, representing 1.77% of the total, experienced a new-onset stroke. The incidence of new-onset stroke differed notably across sleep trajectory groups. For total sleep trajectories, the incidence was 1.4% among steady high sleepers, 2.5% among steady low sleepers, 2.6% among inverted U-shaped sleepers, and 2.9% among U-shaped sleepers (Figure S1E in Multimedia Appendix 2). The pattern observed in nocturnal sleep trajectories (Figure S1F in Multimedia Appendix 2) was similar, with incidences recorded at 2.8%, 2.7%, 1.4%, and 2.7%. Individuals with inverted U-shaped, steady-low, or U-shaped sleep patterns showed an increased risk of new-onset stroke compared to those with consistently high sleep durations. For total sleep duration trajectories, compared with steady high sleepers (reference), individuals in the steady low sleep trajectory exhibited a significantly higher risk of incident stroke in the fully adjusted model (HR=1.71, 95% CI: 1.07‐2.72; *P*=.02; Table 4). In contrast, participants with an inverted U-shaped sleep trajectory showed an elevated but statistically nonsignificant risk of stroke (HR=1.85, 95% CI 0.91‐3.79; *P*=.09). Similarly, no statistically significant association was observed for the U-shaped sleep trajectory (HR=1.77, 95% CI 0.87‐3.60; *P*=.12; Table 4). Figure 3C illustrates the association between total sleep duration and new-onset stroke, with neither the overall association (*P* for overall=.18) nor the nonlinearity test (*P* for nonlinear=.62) reaching statistical significance.

**Table 4.** Association of the sleep trajectories and new-onset stroke.

Characteristic	Total (N)	Event, n (%)	Model 1^a^			Model 2^b^			Model 3^c^		
HR^d^ (95% CI)		*P* value	HR (95% CI)		*P* value	HR (95% CI)		*P* value
Total sleep trajectories	5603	100 (1.78)									
Steady high sleeper	3784	53 (1.4)	Reference^e^			Reference			Reference		
Steady low sleeper	1124	28 (2.5)	1.79 (1.13, 2.83)		.01	1.67 (1.05-2.66)		.03	1.71 (1.07-2.72)		.02
Inverted U-shaped sleeper	349	9 (2.6)	1.85 (0.91-3.75)		.09	1.68 (0.82-3.42)		.16	1.85 (0.91-3.79)		.09
U-shaped sleeper	346	10 (2.9)	2.09 (1.06-4.10)		.03	1.93 (0.97-3.82)		.06	1.77 (0.87-3.60)		.12
Nocturnal sleep trajectories	5603	100 (1.78)									
Steady high sleeper	3849	52 (1.4)	Reference			Reference			Reference		
Steady low sleeper	1073	29 (2.7)	2.02 (1.28-3.18)		.002	1.87 (1.18-2.96)		.008	1.83 (1.15-2.91)		.01
Inverted U-shaped sleeper	386	11 (2.8)	2.12 (1.11-4.06)		.02	1.93 (1.00-3.73)		.05	2.10 (1.08-4.05)		.03
U-shaped sleeper	295	8 (2.7)	2.02 (0.96-4.26)		.06	1.89 (0.89-4.00)		.10	1.96 (0.92-4.18)		.08

a Model 1: crude model.

b Model 2: adjusted for age, gender, education level, marital status, drinking status, and smoking status.

c Model 3: adjusted for age, gender, education level, marital status, drinking status, smoking status, dyslipidemia (current use of lipid-lowering medications, and/or triglycerides>150 mg/dL, high-density lipoprotein cholesterol [HDL-C]<40 mg/dL, low-density lipoprotein cholesterol [LDL-C]>160 mg/dL, or total cholesterol [TC]>240 mg/dL), blood urea nitrogen, estimated glomerular filtration rate, diabetes mellitus, and obesity status.

d HR: hazard ratio.

e Participants with steady high sleep duration, which is used as the reference for comparison when reporting hazard ratios (HR) for the other sleep trajectory groups.

For nocturnal sleep duration trajectories, the associations were more pronounced. Compared with steady high sleepers (reference), individuals in the steady low-sleep trajectory exhibited a significantly increased risk of new-onset stroke in the fully adjusted model (HR=1.83, 95% CI 1.15‐2.91; *P*=.01; Table 4). In contrast, participants with an inverted U-shaped sleep trajectory also showed a significantly elevated risk of incident stroke (HR=2.10, 95% CI 1.08‐4.05; *P*=.03). By comparison, although the U-shaped sleep trajectory was associated with a higher hazard ratio, the association did not reach statistical significance (HR=1.96, 95% CI 0.92‐4.18; *P*=.08). Figure 3G illustrates the association between nocturnal sleep duration and new-onset stroke, with neither the overall association (*P* for overall=0.14) nor the nonlinearity test (*P* for nonlinear=0.84) reaching statistical significance.

### Association of Sleep Duration Trajectories With New-Onset Heart Disease

Over the follow-up duration, 438 participants (7.8%) were found to have newly developed heart disease. The incidence of new-onset heart disease differed modestly across sleep trajectory groups. For total sleep trajectories (Figure S1G in Multimedia Appendix 2), the lowest incidence was observed among steady high sleepers (7.3%), followed by steady low sleepers (8.8%) and U-shaped sleepers (7.8%), whereas inverted U-shaped sleepers exhibited the highest incidence (10.0%). A similar pattern was observed for nocturnal sleep duration trajectories (Figure S1H in Multimedia Appendix 2). The incidence of new-onset heart disease was 7.5% among steady high sleepers, increased to 8.5% among steady low sleepers, and peaked among inverted U-shaped sleepers (8.8%) before declining in the U-shaped group (7.8%). For total sleep duration trajectories, compared with steady high sleepers (reference), no statistically significant associations were observed across trajectory groups in the fully adjusted model. Specifically, the steady low sleep trajectory was associated with a modestly elevated but nonsignificant risk of heart disease (HR=1.14, 95% CI 0.90‐1.43; *P*=.27; Table 5). Similarly, participants with an inverted U-shaped sleep trajectory showed an increased HR that did not reach statistical significance (HR=1.34, 95% CI: 0.94‐1.90; *P*=.11). The U-shaped sleep trajectory was not associated with heart disease risk (HR=0.93, 95% CI 0.63‐1.39; *P*=.73). Figure 3D illustrates the association between total sleep duration and heart disease risk. The overall association reached statistical significance (*P* for overall=.03); however, there was no evidence supporting a nonlinear relationship (*P* for nonlinear=.45).

**Table 5.** Association of the sleep trajectories and new-onset heart disease.

Characteristic	Total (N)	Event, n (%)	Model 1^a^			Model 2^b^			Model 3^c^		
			HR^d^ (95% CI)		*P* value	HR (95% CI)		*P* value	HR (95% CI)		*P* value
Total sleep trajectories	5603	438 (7.82)									
Steady high sleeper	3784	276 (7.3)	Reference^e^			Reference			Reference		
Steady low sleeper	1124	99 (8.8)	1.22 (0.97-1.53)		.09	1.11 (0.88-1.40)		.36	1.14 (0.90-1.43)		.29
Inverted U-shaped sleeper	349	36 (10)	1.44 (1.02-2.04)		.04	1.27 (0.89-1.80)		.18	1.34 (0.94-1.90)		.11
U-shaped sleeper	346	27 (7.8)	1.07 (0.72-1.59)		.75	0.95 (0.64-1.41)		.79	0.93 (0.63-1.39)		.73
Nocturnal sleep trajectories	5603	438 (7.82)									
Steady high sleeper	3849	290 (7.5)	Reference			Reference			Reference		
Steady low sleeper	1073	91 (8.5)	1.13 (0.89-1.43)		.30	1.04 (0.82-1.32)		.72	1.06 (0.84-1.35)		.62
Inverted U-shaped sleeper	386	34 (8.8)	1.19 (0.83-1.69)		.35	1.04 (0.73-1.49)		.83	1.09 (0.76-1.56)		.63
U-shaped sleeper	295	23 (7.8)	1.03 (0.67-1.57)		.90	0.91 (0.60-1.40)		.68	0.91 (0.60-1.40)		.68

a Model 1: crude model.

b Model 2: adjusted for age, gender, education level, marital status, drinking status, and smoking status.

c Model 3: adjusted for age, gender, education level, marital status, drinking status, smoking status, dyslipidemia (current use of lipid-lowering medications, and/or triglycerides>150 mg/dL, high-density lipoprotein cholesterol [HDL-C]<40 mg/dL, low-density lipoprotein cholesterol [LDL-C]>160 mg/dL, or total cholesterol [TC]>240 mg/dL), blood urea nitrogen, estimated glomerular filtration rate, diabetes mellitus, and obesity status.

d HR: hazard ratio.

e Participants with steady high sleep duration, which is used as the reference for comparison when reporting hazard ratios (HR) for the other sleep trajectory groups.

### Sensitivity Analysis

In the sensitivity analysis excluding participants who developed hypertension within the first 2 years of follow-up (Table S3 in Multimedia Appendix 1), compared with steady high sleepers (reference), individuals with an inverted U-shaped sleep trajectory exhibited a significantly increased risk in the fully adjusted total sleep trajectory model (HR=1.34, 95% CI 1.06‐1.71; *P*=.02). In contrast, compared with the steady high sleepers (reference), the association remained statistically significant in the fully adjusted nocturnal sleep trajectory model (HR=1.28, 95% CI 1.02‐1.62; *P*=.03; Table S3 in Multimedia Appendix 1). In the sensitivity analysis excluding early CVD within the first 2 years of follow-up (Table S4 in Multimedia Appendix 1), the association between the inverted U-shaped total sleep trajectory and incident CVD was weakened and did not reach statistical significance after full adjustment (HR=1.38, 95% CI 0.93‐2.04; *P*=.11). Excluding strokes that occurred within the first 2 years of follow-up (Table S5 in Multimedia Appendix 1), the association for total sleep trajectories was attenuated. Specifically, although individuals with an inverted U-shaped total sleep trajectory showed an elevated HR for incident stroke, the association did not reach statistical significance in the fully adjusted model (HR=2.23, 95% CI 0.90‐5.51; *P*=.08). In contrast, for nocturnal sleep trajectories, the inverted U-shaped sleep pattern remained robustly and independently associated with an increased risk of new-onset stroke after full adjustment (HR=2.91, 95% CI 1.28‐6.61; *P*=.01).

In misclassification-adjusted analyses using posterior-probability-weighted Cox regression (Table S6A and S6B in Multimedia Appendix 1), the overall patterns of association were largely consistent with those observed in the primary analyses. In particular, the inverted U-shaped sleep trajectory remained associated with an increased risk of adverse CVD including stroke (Total sleep trajectories-CVD- HR=1.47, 95% CI 1.07‐2.02; *P*=.02; Nocturnal sleep trajectories-stroke-HR=1.29, 95% CI: 1.06‐2.71, *P*=.03; Table S6B in Multimedia Appendix 1) and hypertension (Total sleep trajectories: HR=1.35, 95% CI 1.07‐1.69; *P*=.01; Table S6A in Multimedia Appendix 1), although the strength and statistical significance of the associations varied across specific outcomes.

In sensitivity analyses with additional adjustment for SES <4 (Table S7A and S7B in Multimedia Appendix 1), the overall patterns of association between sleep trajectories and cardiometabolic outcomes were largely consistent with the primary analyses. Specifically, the inverted U-shaped pattern of total sleep maintained a significant association with a higher risk of new CVD (HR=1.58, 95% CI: 1.06‐2.34; *P*=.02), but the link with hypertension was weakened after SES adjustment (HR=1.45, 95% CI 1.09‐1.93; *P*=.01; Table S7A in Multimedia Appendix 1). In the same socioeconomic stratum, among nocturnal sleep trajectories, a significant association was observed between the inverted U-shaped nocturnal sleep pattern and incident stroke among participants with lower SES of <4 (HR=3.07, 95% CI 1.24‐7.58; *P*=.01; Table S7B in Multimedia Appendix 1).

### Mediator Analysis

**Figure 4.**
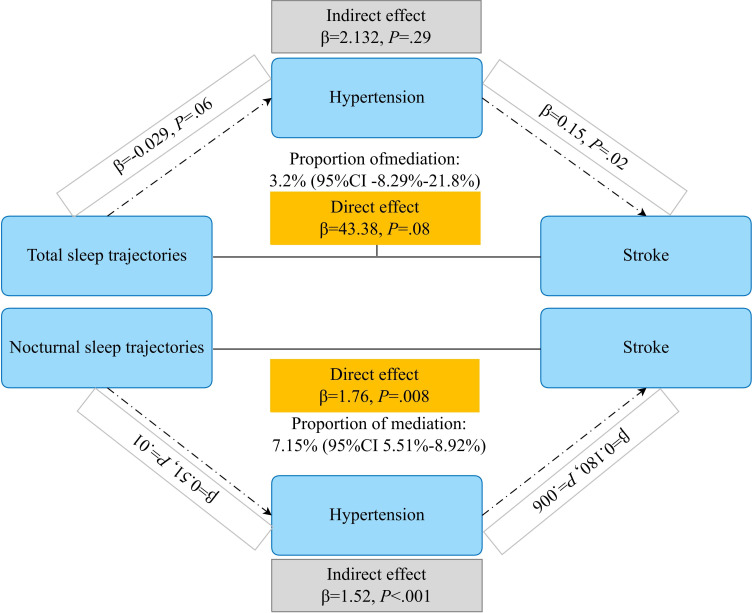
Mediation analysis of the association between the total sleep/nocturnal sleep duration trajectories and stroke.

Mediation analyses were conducted to assess whether hypertension mediated the associations between sleep duration trajectories and incident CVD, stroke, and heart disease. For total sleep duration trajectories, the indirect effect mediated through hypertension was not statistically significant (*β*=2.13; *P*=.30), accounting for 3.2% of the total effect (95% CI −8.29% to 21.8%). The direct effect of total sleep trajectories on stroke risk was also not statistically significant (*β*=43.38; *P*=.08; Figure 4). In contrast, for nocturnal sleep duration trajectories, a significant indirect effect through hypertension was observed (*β*=1.52; *P*<.001), with hypertension mediating 7.15% of the total association between nocturnal sleep trajectories and stroke risk (95% CI 5.51%‐8.92%; Figure 4). Notably, the direct effect of nocturnal sleep trajectories on stroke remained statistically significant after accounting for hypertension (*β*=1.76; *P*=.008; Figure 4). Hypertension partially mediated the association between sleep duration trajectories and stroke. For total sleep duration trajectories, neither the indirect effect through hypertension nor the direct effect on CVD reached statistical significance (indirect effect: *β*=0.050; *P*=.37; direct effect: *β*=0.029; *P*=.09), despite a modest estimated mediation proportion (8.7%; Figure S2 in Multimedia Appendix 2). Similar null findings were observed for nocturnal sleep duration trajectories, with nonsignificant indirect and direct effects on CVD. Consistent results were observed for heart disease, where neither total nor nocturnal sleep duration trajectories showed significant indirect effects mediated by hypertension, and all corresponding direct effects were also nonsignificant (Figure 3 in Multimedia Appendix 2).

## Discussion

### Principal Findings

In this population-based study that is both longitudinal and nationally representative, researchers identified 4 distinct trajectories of total and nocturnal sleep duration. It was found that an inverted U-shaped sleep pattern was related to a greater risk of CVD, irrespective of typical risk factors. Multiple sensitivity analyses showed that these associations remained largely stable, supporting the strength of the results. We also explored the potential role of hypertension as an intermediate factor and observed preliminary evidence suggesting that hypertension may partly account for the relationship between unstable sleep trajectories and incident CVD.

This analysis highlights a clear inverted U-shaped association between sleep amount and the chance of developing hypertension. The risk of hypertension is considerably higher in individuals with an inverted U-shaped sleep duration trajectory than in those who consistently sleep for extended periods. In Model 3, the inverted U-shaped total sleep trajectory was associated with an increased risk of hypertension (HR=1.34, 95% CI 1.07‐1.69; *P*=.01) [28]. In comparison, groups with a consistently low amount of sleep and those with a U-shaped sleep pattern did not have a significant association with the risk of hypertension. The important link identified in the inverted U-shape group is in line with past research that has related irregular or disrupted sleep patterns to an increased likelihood of hypertension. The nonlinear relationship between total sleep time and hypertension risk indicates that variability in sleep duration, rather than consistently short or long sleep, might be an important factor in the onset of hypertension. This association may be partly explained by the physiological stress induced by unstable sleep patterns, which can contribute to dysregulation of the autonomic nervous system, heightened inflammatory responses, and disruptions of circadian rhythms, all of which have been implicated in the development of hypertension [29].

With regard to the development of new-onset CVD, our findings indicate individuals with inverted U-shaped sleep duration trajectories exhibited a significantly elevated CVD risk. In the fully adjusted Model 3, the inverted U-shaped trajectory group showed an HR of 1.46 (95 %CI 1.07‐2.00; *P*=.02) for total sleep duration, indicating a trend toward increased risk. Conversely, the U-shaped and steady low trajectory showed no significant association, likely due to limited statistical power from a smaller sample size. These results are consistent with meta-analytic data, which consistently show an irregular or insufficient relationship between sleep duration and CVD risk, with optimal cardiovascular outcomes occurring between 7 and 8 hours of sleep per night [30]. The underlying physiological mechanisms for these associations likely involve multiple factors. Short sleep may increase stress hormone levels and inflammation, leading to endothelial dysfunction and atherosclerosis [29], while long sleep durations may be indicative of comorbid conditions such as depression or metabolic syndrome, both of which are known to be significant risk factors for CVD [30]. The study further suggests that individuals with erratic sleep patterns over the long term have a substantially higher risk of CVD, even when their sleep duration falls within the recommended range, emphasizing the role of sleep variability in cardiovascular risk.

In terms of stroke risk, there was a significant connection between the duration of sleep at night and the onset of new strokes in those with an inverted U-shaped sleep pattern, with a HR of 2.10 (95% CI 1.08‐4.05; *P*=.03). This observation is consistent with findings from large cohort studies that have identified short sleep as a key risk factor for stroke, likely due to its impact on raising blood pressure and altering circadian rhythms [28,30]. The steady low sleep trajectory was associated with a significantly increased risk of stroke compared with the steady high sleep trajectory, both for total sleep duration (HR=1.71, 95% CI 1.07‐2.72; *P*=.02) and nocturnal sleep duration (HR=1.83, 95% CI 1.15‐2.91; *P*=.01). The evidence points to the possibility that chronic sleep disruption, either from insufficient sleep or prolonged sleeping, may heighten the likelihood of stroke. However, after excluding strokes occurring within the first 2 years of follow-up, the steady low sleep trajectory remained significantly associated with stroke risk, supporting the robustness of the observed association in longer-term follow-up.

No significant relationships were found between new-onset heart disease and any sleep duration trajectories, whether for overall sleep or night sleep duration. Unlike the associations seen with hypertension, CVD, and stroke, this suggests that the link between sleep duration and heart disease could differ from other cardiovascular conditions. Underlying health issues or genetic factors could potentially have a greater influence on the development of heart disease. In addition, the insignificant correlation may result from the relatively low prevalence of heart disease in our study population, highlighting the importance of conducting future research with more extensive sample sizes and prolonged follow-up durations.

Furthermore, our exploratory mediation analysis indicated that hypertension was responsible for only a slight portion of the association between nocturnal sleep patterns and stroke, comprising a modest fraction of the overall effect. The pattern suggests a potential relationship between sleep habits and stroke risk, but the temporal structure of the data does not allow for a definitive causal interpretation. No notable mediation was detected for CVD or heart disease, which suggests that the association between sleep and cardiovascular health might operate through mechanisms other than hypertension. Overall, these findings are consistent with the possibility that sleep health influences cardiovascular risk partly through its relationship with blood pressure regulation, although confirmation will require longitudinal data with more precise temporal ordering [31].

These findings are consistent with previous research linking irregular or insufficient sleep to negative cardiovascular outcomes, although the lack of significant associations for heart disease in Table 5 suggests that the effects may be specific to certain outcomes. The inverted U-shaped sleep duration trajectory consistently showed the strongest associations across outcomes, particularly for stroke, with a HR of 2.10 for nocturnal sleep. These findings are consistent with existing evidence suggesting that chronic sleep deprivation may contribute to endothelial dysfunction, oxidative stress, and inflammation, which are key mechanisms underlying cardiovascular and cerebrovascular pathogenesis. The borderline associations between the inverted U-shaped sleep trajectory and CVD and stroke may indicate potential involvement of circadian disruption or heightened sympathetic activity, whereas the nonsignificant findings for heart disease could be attributable to reduced statistical power or heterogeneity in disease pathophysiology. The absence of significant associations for the U-shaped sleep trajectory across outcomes may suggest that compensatory increases in sleep duration following periods of reduction could partially offset adverse effects. However, this hypothesis warrants further investigation in larger cohorts. The observed significant associations, especially for steady low sleepers, persisted after extensive adjustment for demographic, lifestyle, and clinical confounders, supporting the robustness of the results.

Identifying harmful trends in sleep duration has important implications for public health policies and medical practices. From the viewpoint of population health, those who regularly experience short, long, or erratic sleep durations form a high-risk group. Observing their sleep patterns over time could aid in early preventive interventions. Longitudinal data reveal that unhealthy sleep trends are associated with a heightened risk of cardiovascular conditions and death [32]. For example, a recent cohort study revealed that inconsistent sleep duration over many years significantly elevated the risk of cardiovascular problems [33]. Thus, ongoing monitoring of sleep duration and its time-related trends might contribute to risk assessment and efforts to reduce cardiovascular burden on a population scale. In a clinical context, recognizing distinct sleep-duration trajectories supports the inclusion of sleep pattern assessments in regular cardiovascular risk evaluations. Incorporating sleep-trajectory data alongside conventional risk factors (hypertension, dyslipidemia, and obesity) enables targeted interventions, including behavioral counseling on sleep hygiene, cognitive behavioral therapy for insomnia, and wearable-device monitoring, with the aim of stabilizing trajectories and mitigating subsequent cardiometabolic risk. From a mechanistic standpoint, ongoing deviations from normal sleep patterns could amplify pathophysiological processes such as sympathetic overactivity, chronic inflammation, and endothelial dysfunction, which are involved in the progression of CVD [34-36]. Further studies should integrate objective sleep-monitoring tools, such as actigraphy or polysomnography, with precision medicine approaches to enhance risk prediction and investigate if modifying detrimental sleep patterns affects cardiovascular health.

The strengths of the current study lie in the large, nationally representative sample used for the analysis, which enhances the generalizability of the findings. Additionally, the study results remained robust across several sensitivity analyses, which further supports the reliability of the findings. The use of LCMM allowed for the clustering of individuals with similar underlying trajectories, minimizing potential misclassification and providing a more accurate representation of sleep duration patterns over time. However, several limitations must be acknowledged. First, as an observational study, the inability to establish causality is a fundamental limitation. Despite controlling for numerous potential confounders, residual confounding due to unmeasured variables cannot be ruled out, and the nonrandomized nature of the data further complicates causal inference. Although we excluded participants with prevalent CVD prior to the follow-up period and conducted a value analysis to address this concern, the observational design still precludes definitive causal conclusions. Second, the research was based on self-reported data for baseline measurements, particularly in terms of sleep duration and health outcomes, which might introduce recall bias or misclassification. The relevance of this bias is heightened among older adults, whose cognitive or memory challenges may compromise the accuracy of their self-reported information. Misreporting could result in nondifferential measurement errors, weakening the associations between sleep duration trajectories and cardiovascular outcomes, and possibly impacting the precision of trajectory classification. Nonetheless, self-reported sleep duration has been widely confirmed and applied in large-scale epidemiological studies like CHARLS, and it still serves as a practical and reliable measure for research on populations. To substantiate these findings and delve deeper into the connection between sleep patterns and cardiovascular health, future research should incorporate objective measures such as actigraphy or polysomnography. Third, although residual confounding cannot be fully ruled out, CHARLS lacks detailed dietary data, and the physical activity information has significant missing elements that could introduce bias if incorporated into the models. When interpreting the current findings, these data constraints should be taken into account. Fourth, due to the study population consisting of older adults who survived to the baseline, survivor bias might have occurred, leading to an underrepresentation of those with severe comorbidities or early death, which may have lessened the impact of the findings. A thorough examination of bias suggests that selection bias, information bias, and residual confounding together may have slightly influenced the effect estimates, underscoring the importance of careful interpretation. Fifth, the absence of cause-specific mortality records in the CHARLS dataset makes it impossible to identify whether deaths were due to cardiovascular or noncardiovascular causes. Consequently, the application of competing-risk models was not possible, possibly affecting the precision of cardiovascular event risk estimation. Finally, the sample comprised primarily middle-aged and older Chinese adults, limiting the external validity of the findings. The results may not be fully generalizable to younger populations or individuals from other countries or ethnic groups. Future research should seek to replicate these findings in more diverse populations and consider additional factors, such as sleep quality, sleep disorders, and other contextual variables, which may further refine our understanding of the relationship between sleep duration and cardiovascular health.

### Conclusions

Our findings indicate that the longitudinal stability of sleep duration is an important marker of cardiovascular health. Sleep trajectories with continuous insufficiency or marked fluctuations were related to higher chances of negative cardiovascular events. In exploratory mediation analyses, hypertension accounted for a small proportion of the association between nocturnal sleep trajectories and stroke, suggesting a possible pathway through which sleep disruption may influence cerebrovascular risk. These results highlight the potential relevance of maintaining consistent sleep patterns as part of strategies to support cardiovascular health, while underscoring the need for future longitudinal research with clearer temporal ordering to clarify underlying mechanisms.

## Supplementary material

10.2196/78914 Multimedia Appendix 1 Supplementary tables on latent class mixed model fit indices, sensitivity analyses, misclassification-adjusted Cox regression analyses, and socioeconomic status–adjusted associations between sleep trajectories and incident hypertension, cardiovascular disease (CVD), stroke, and heart disease.

10.2196/78914 Multimedia Appendix 2 Supplementary figures showing new-onset rates of hypertension, cardiovascular disease (CVD), stroke, and heart disease according to sleep duration trajectories, and mediation analyses of the associations between sleep trajectories and CVD/heart disease.

## Abbreviations

BUN: blood urea nitrogen
CHARLS: China Health and Retirement Longitudinal Study
CVD: cardiovascular disease
DBP: diastolic blood pressure
DM: diabetes mellitus
eGFR: estimated glomerular filtration rate
FPG: fasting plasma glucose
HbA_1c_: hemoglobin A_1c_
HR: hazard ratio
LCMM: latent class mixed models
MICE: multiple imputation by chained equation
SBP: systolic blood pressure
SES: socioeconomic status
STROBE: Strengthening the Reporting of Observational Studies in Epidemiology

## References

[1] World Health Organization (2020). UN Decade of Healthy Ageing: Plan of Action 2021–2030.

[2] World Health Organization (2015). China country assessment report on ageing and health. World Health Organization.

[3] North BJ, Sinclair DA (2012). The intersection between aging and cardiovascular disease. Circ Res.

[4] Buford TW (2016). Hypertension and aging. Ageing Res Rev.

[5] Yousufuddin M, Young N (2019). Aging and ischemic stroke. Aging (Albany NY).

[6] Lakatta EG, Levy D (2003). Arterial and cardiac aging: major shareholders in cardiovascular disease enterprises: part II: the aging heart in health: links to heart disease. Circulation.

[7] Ford ES (2011). Trends in mortality from all causes and cardiovascular disease among hypertensive and nonhypertensive adults in the United States. Circulation.

[8] Chattu VK, Manzar MD, Kumary S, Burman D, Spence DW, Pandi-Perumal SR, Kumary S (2018). The global problem of insufficient sleep and its serious public health implications. Healthcare (Basel).

[9] Dong L, Xie Y, Zou X (2022). Association between sleep duration and depression in US adults: a cross-sectional study. J Affect Disord.

[10] Cai R, Chao J, Gao C, Gao L, Hu K, Li P (2025). Association between sleep duration and cognitive frailty in older Chinese adults: prospective cohort study. JMIR Aging.

[11] Erren TC, Morfeld P, Foster RG, Reiter RJ, Groß JV, Westermann IK (2016). Sleep and cancer: synthesis of experimental data and meta-analyses of cancer incidence among some 1,500,000 study individuals in 13 countries. Chronobiol Int.

[12] Ungvari Z, Fekete M, Varga P (2025). Imbalanced sleep increases mortality risk by 14-34%: a meta-analysis. Geroscience.

[13] Wang D, Li W, Cui X (2016). Sleep duration and risk of coronary heart disease: a systematic review and meta-analysis of prospective cohort studies. Int J Cardiol.

[14] Li W, Wang D, Cao S (2016). Sleep duration and risk of stroke events and stroke mortality: a systematic review and meta-analysis of prospective cohort studies. Int J Cardiol.

[15] Meng L, Zheng Y, Hui R (2013). The relationship of sleep duration and insomnia to risk of hypertension incidence: a meta-analysis of prospective cohort studies. Hypertens Res.

[16] Zhao Y, Hu Y, Smith JP, Strauss J, Yang G (2014). Cohort profile: the China Health and Retirement Longitudinal Study (CHARLS). Int J Epidemiol.

[17] Fang J, Wen Z, Ouyang J, Wang H (2021). Modeling the change trajectory of sleep duration and its associated factors: based on an 11-year longitudinal survey in China. BMC Public Health.

[18] Yang M, Liu J, Shen Q (2024). Body roundness index trajectories and the incidence of cardiovascular disease: evidence from the China health and retirement longitudinal study. J Am Heart Assoc.

[19] Zhang Z, Zhao L, Lu Y, Xiao Y, Zhou X (2024). Insulin resistance assessed by estimated glucose disposal rate and risk of incident cardiovascular diseases among individuals without diabetes: findings from a nationwide, population based, prospective cohort study. Cardiovasc Diabetol.

[20] Liu H, Xu F, Zhang M, Chen X, Fan H, Hou M (2025). Association between body roundness index and new-onset stroke risk in middle-aged and older adults with varying glucose metabolism status: a longitudinal study using CHARLS data. Diabetes Res Clin Pract.

[21] Williams B, Mancia G, Spiering W (2018). 2018 ESC/ESH guidelines for the management of arterial hypertension. Eur Heart J.

[22] Yu J, Yi Q, Chen G (2022). The visceral adiposity index and risk of type 2 diabetes mellitus in China: a national cohort analysis. Diabetes Metab Res Rev.

[23] Zhou Z, Ong KL, Whelton SP (2022). Impact of blood lipids on 10-year cardiovascular risk in individuals without dyslipidemia and with low risk factor burden. Mayo Clin Proc.

[24] Zheng X, Han L, Shen S (2022). Hypertension, remnant cholesterol and cardiovascular disease: evidence from the China health and retirement longitudinal study. J Hypertens.

[25] Pan XF, Wang L, Pan A (2021). Epidemiology and determinants of obesity in China. Lancet Diabetes Endocrinol.

[26] Li C, Ma Y, Yang C, Hua R, Xie W, Zhang L (2022). Association of cystatin C kidney function measures with long-term deficit-accumulation frailty trajectories and physical function decline. JAMA Netw Open.

[27] Li C, He D, Liu Y, Yang C, Zhang L (2025). Anti-hypertensive medication adherence, socioeconomic status, and cognitive aging in the Chinese community-dwelling middle-aged and older adults ≥ 45 years: a population-based longitudinal study. BMC Med.

[28] Han B, Chen WZ, Li YC, Chen J, Zeng ZQ (2020). Sleep and hypertension. Sleep Breath.

[29] Gangwisch JE, Malaspina D, Boden-Albala B, Heymsfield SB (2005). Inadequate sleep as a risk factor for obesity: analyses of the NHANES I. Sleep.

[30] Zhao Y, Li J, Li Y (2022). Sleep duration and risk of cardio-cerebrovascular disease: A meta-analysis. Front Cardiovasc Med.

[31] Li J, Li Y, Li Y (2023). Relationship of sleep duration with incident cardiovascular outcomes: a cohort study. BMC Public Health.

[32] Zhang J, Chen L, Zhang S (2023). Associations of sleep patterns with dynamic trajectory of cardiovascular multimorbidity and mortality: a multistate analysis of a large cohort. J Am Heart Assoc.

[33] Thurston RC, Chang Y, Kline CE (2024). Trajectories of sleep over midlife and incident cardiovascular disease events in the study of women’s health across the nation. Circulation.

[34] Greenlund IM, Carter JR (2022). Sympathetic neural responses to sleep disorders and insufficiencies. Am J Physiol Heart Circ Physiol.

[35] Irwin MR (2019). Sleep and inflammation: partners in sickness and in health. Nat Rev Immunol.

[36] Calvin AD, Covassin N, Kremers WK (2014). Experimental sleep restriction causes endothelial dysfunction in healthy humans. J Am Heart Assoc.

