# Supplementary material for: Subsequent Hypertension’s Mediation of the Association Between Sleep Duration Trajectories and New-Onset Cardiovascular Disease: Population-Based Cohort Study
Source: JMIR Aging. 2026 May 28;9:e78914. doi: 10.2196/78914 (PMC13218652; doi:10.2196/78914)
Supplement: Multimedia Appendix 1 [file aging-v9-e78914-s001.docx]

**Subsequent hypertension mediates the association between sleep duration trajectories and new-onset cardiovascular disease: a population-based cohort study**

**Table S1** Fit indices of latent class mixed model on total sleep duration trajectories

**Table S2** Fit indices of latent class mixed model on nocturnal sleep duration trajectories

**Table S3** Association of the total sleep trajectories and new-onset hypertension by excluding hypertension occurring within the first two years

**Table S4** Association of the total sleep trajectories and new-onset CVD by excluding CVD occurring within the first two years

**Table S5** Association of the sleep trajectories and new-onset stroke by excluding stroke occurring within the first two years

**Table S6A** Misclassification-adjusted Cox regression for incident hypertension and CVD based on posterior-probability weighting

**Table S6B** Misclassification-adjusted Cox regression for incident stroke and heart disease based on posterior-probability weighting

**Table S7A** Association of the total/nocturnal sleep trajectories and new-onset hypertension/CVD after additional adjustment for socioeconomic status

**Table S7B** Association of the total/nocturnal sleep trajectories and new-onset stroke/heart disease after additional adjustment for socioeconomic status

**Table S1** Fit indices of the latent class mixed model on total sleep duration trajectories

| **Model** | **Log likelihood** | **AIC** | **BIC** | **aBIC** | **Entropy** | **Class (%)** |
| --- | --- | --- | --- | --- | --- | --- |
| 1 | -7231.86 | 14499.72 | 14619.28 | 14562.08 | 1.00 | 100 |
| 2 | -7107.33 | 13890.84 | 14103.39 | 14001.71 | 0.33 | 27.46 / 72.53 |
| 3 | -6710.25 | 13512.50 | 13818.04 | 13671.87 | 0.56 | 16.18/ 74.75/ 9.07 |
| 4 | -6532.59 | 13185.17 | 13583.72 | 13393.06 | 0.59 | 7.04 / 21.17 / 65.62 / 6.15 |
| 5 | -5572.69 | 11293.38 | 11784.92 | 11549.77 | 0.57 | 11.58/4.01/ 38.13/37.48 / 8.81 |
| 6 | -5511.04 | 11198.08 | 11782.61 | 11502.97 | 0.56 | 6.97/ 33.30 /14.29 / 36.63 /21.3 /5.2 |

**Abbreviations:** AIC, Akaike Information Criterion; BIC, Bayesian Information Criterion; aBIC, adjusted Bayesian Information Criterion.

**Table S2** Fit indices of latent class mixed model on nocturnal sleep duration trajectories

| **Model** | **Log likelihood** | **AIC** | **BIC** | **aBIC** | **Entropy** | **Class (%)** |
| --- | --- | --- | --- | --- | --- | --- |
| 1 | -6556.61 | 13149.22 | 13268.78 | 13211.58 | 1.00 | 100 |
| 2 | -6133.27 | 12330.54 | 12543.09 | 12441.41 | 0.38 | 28.87 / 71.13 |
| 3 | -5873.37 | 11838.73 | 12144.29 | 11998.11 | 0.59 | 15.88/ 73.30/ 10.82 |
| 4 | -5665.96 | 11451.92 | 11850.46 | 11659.80 | 0.73 | 7.48 / 20.70 / 66.56/ 5.26 |
| 5 | -6532.59 | 13185.17 | 13587.81 | 13393.06 | 0.60 | 4.3 /5.9/ 66.5/20.5 / 2.9 |
| 6 | -6607.16 | 13390.32 | 13616.48 | 13696.86 | 0.50 | 6.9/ 3.7 / 25.0 / 37.9 /4.41 /4.39 |

**Abbreviations:** AIC, akaike information criterion; BIC, Bayesian information criterion; aBIC, adjusted Bayesian information criterion

**Table S3** Association of the total sleep trajectories and new-onset hypertension by excluding hypertension occurring within the first two year

| **Characteristic** | **N** | **Event N** | **Model 1** | | | **Model 2** | | | **Model 3** | | |
| --- | --- | --- | --- | --- | --- | --- | --- | --- | --- | --- | --- |
|  |  |  | HR | 95% CI | **p-value** | HR | 95% CI | **p-value** | HR | 95% CI | **p-value** |
| **Total sleep trajectories** | 5,498 | 1,018 |  | | |  | | |  | | |
| steady high sleeper | 3,713 | 655 (18%) | Reference | Reference |  | Reference | Reference |  | Reference | Reference |  |
| steady low sleeper | 1,102 | 220 (20%) | 1.15 | 0.98, 1.33 | 0.08 | 1.12 | 0.96, 1.30 | 0.16 | 1.14 | 0.97, 1.33 | 0.10 |
| **inverted U-shape sleeper** | 340 | 76 (22%) | 1.30 | 1.03, 1.65 | 0.03 | 1.25 | 0.99, 1.59 | 0.06 | 1.34 | 1.06, 1.71 | 0.02 |
| U-shape sleeper | 343 | 67 (20%) | 1.12 | 0.87, 1.44 | 0.392 | 1.09 | 0.84, 1.40 | 0.51 | 1.04 | 0.80, 1.34 | 0.77 |
| **Nocturnal sleep trajectories** | 5,498 | 1,018 |  |  |  |  |  |  |  |  |  |
| steady high sleeper | 3,776 | 671 (18%) | Reference | Reference |  | Reference | Reference |  | Reference | Reference |  |
| steady low sleeper | 1,052 | 203 (19%) | 1.10 | 0.94, 1.28 | 0.26 | 1.07 | 0.91, 1.25 | 0.41 | 1.07 | 0.91, 1.25 | 0.42 |
| inverted U-shape sleeper | 378 | 83 (22%) | 1.26 | 1.00, 1.58 | 0.05 | 1.20 | 0.96, 1.51 | 0.12 | 1.28 | 1.02, 1.62 | 0.03 |
| U-shape sleeper | 292 | 61 (21%) | 1.19 | 0.92, 1.55 | 0.19 | 1.16 | 0.89, 1.50 | 0.28 | 1.15 | 0.88, 1.49 | 0.31 |

**Model 1:** crude model; **Model 2:** Adjusted for age, gender, education level, marital status, drinking status, and smoking status; **Model 3:** Adjusted for age, gender, education level, marital status, drinking status, smoking status, dyslipidemia (current use of lipid-lowering medications, and/or TG>150 mg/dL, HDL-C<40 mg/dL, LDL-C>160mg/dL, or TC>240mg/dL), BUN, eGFR, DM, and obesity status.

**Abbreviation:** HR, hazard ratio, CI, confidence interval; BUN, blood urea nitrogen; eGFR, estimated glomerular filtration rate; DM, Diabetes mellitus; TG, triglycerides; HDL-C, high-density lipoprotein cholesterol; LDL-C, low-density lipoprotein cholesterol; TC, total cholesterol

**Table S4** Association of the total sleep trajectories and new-onset CVD by excluding CVD occurring within the first two years

| **Characteristic** | **N** | **Event N** | **Model 1** | | | **Model 2** | | | **Model 3** | | |
| --- | --- | --- | --- | --- | --- | --- | --- | --- | --- | --- | --- |
|  |  |  | HR | 95% CI | **p-value** | HR | 95% CI | **p-value** | HR | 95% CI | **p-value** |
| **Total sleep trajectories** | 5,428 | 347 |  | | |  | | |  | | |
| steady high sleeper | 3,678 | 213 (5.8%) | Reference | Reference |  | Reference | Reference |  | Reference | Reference |  |
| steady low sleeper | 1,083 | 81 (7.5%) | 1.31 | 1.01, 1.69 | 0.04 | 1.19 | 0.92, 1.55 | 0.18 | 1.21 | 0.94, 1.57 | 0.14 |
| inverted U-shape sleeper | 333 | 29 (8.7%) | 1.53 | 1.04, 2.26 | 0.03 | 1.34 | 0.91, 1.98 | 0.14 | 1.38 | 0.93, 2.04 | 0.11 |
| U-shape sleeper | 334 | 24 (7.2%) | 1.25 | 0.82, 1.90 | 0.31 | 1.08 | 0.71, 1.66 | 0.71 | 1.07 | 0.70, 1.64 | 0.76 |
| **Nocturnal sleep trajectories** | 5,428 | 347 |  |  |  |  |  |  |  |  |  |
| steady high sleeper | 3,741 | 221 (5.9%) | Reference | Reference |  | Reference | Reference |  | Reference | Reference |  |
| steady low sleeper | 1,032 | 76 (7.4%) | 1.26 | 0.97, 1.64 | 0.08 | 1.16 | 0.89, 1.51 | 0.28 | 1.18 | 0.90, 1.54 | 0.22 |
| inverted U-shape sleeper | 368 | 27 (7.3%) | 1.26 | 0.85, 1.88 | 0.26 | 1.10 | 0.74, 1.65 | 0.66 | 1.14 | 0.76, 1.72 | 0.51 |
| U-shape sleeper | 287 | 23 (8.0%) | 1.37 | 0.89, 2.10 | 0.15 | 1.20 | 0.78, 1.86 | 0.40 | 1.21 | 0.78, 1.86 | 0.40 |

**Model 1:** crude model; **Model 2:** Adjusted for age, gender, education level, marital status, drinking status, and smoking status; **Model 3:** Adjusted for age, gender, education level, marital status, drinking status, smoking status, dyslipidemia (current use of lipid-lowering medications, and/or TG>150 mg/dL, HDL-C<40 mg/dL, LDL-C>160mg/dL, or TC>240mg/dL), BUN, eGFR, DM, and obesity status.

**Abbreviation:** HR, hazard ratio, CI, confidence interval; BUN, blood urea nitrogen; eGFR, estimated glomerular filtration rate; DM, Diabetes mellitus; TG, triglycerides; HDL-C, high-density lipoprotein cholesterol; LDL-C, low-density lipoprotein cholesterol; TC, total cholesterol

**Table S5** Association of the sleep trajectories and new-onset stroke by excluding stroke occurring within the first two years

| **Characteristic** | **N** | **Event N** | **Model 1** | | | **Model 2** | | | **Model 3** | | |
| --- | --- | --- | --- | --- | --- | --- | --- | --- | --- | --- | --- |
|  |  |  | HR | 95% CI | **p-value** | HR | 95% CI | **p-value** | HR | 95% CI | **p-value** |
| **Total sleep trajectories** | 5,549 | 46 |  | | |  | | |  | | |
| steady high sleeper | 3,756 | 25 (0.7%) | Reference | Reference |  | Reference | Reference |  | Reference | Reference |  |
| steady low sleeper | 1,108 | 12 (1.1%) | 1.63 | 0.82, 3.25 | 0.16 | 1.41 | 0.70, 2.82 | 0.34 | 1.43 | 0.71, 2.87 | 0.32 |
| inverted U-shape sleeper | 346 | 6 (1.7%) | 2.62 | 1.08, 6.39 | 0.03 | 2.09 | 0.85, 5.15 | 0.11 | 2.23 | 0.90, 5.51 | 0.08 |
| U-shape sleeper | 339 | 3 (0.9%) | 1.33 | 0.40, 4.41 | 0.64 | 1.05 | 0.31, 3.52 | 0.94 | 0.97 | 0.28, 3.35 | 0.96 |
| **Nocturnal sleep trajectories** | 5,549 | 46 |  |  |  |  |  |  |  |  |  |
| steady high sleeper | 3,820 | 23 (0.6%) | Reference | Reference |  | Reference | Reference |  | Reference | Reference |  |
| steady low sleeper | 1,056 | 12 (1.1%) | 1.89 | 0.94, 3.81 | 0.07 | 1.66 | 0.82, 3.36 | 0.16 | 1.57 | 0.77, 3.19 | 0.21 |
| **inverted U-shape sleeper** | 383 | 8 (2.1%) | 3.49 | 1.56, 7.81 | 0.002 | 2.86 | 1.26, 6.49 | 0.01 | 2.91 | 1.28, 6.61 | 0.01 |
| U-shape sleeper | 290 | 3 (1.0%) | 1.72 | 0.52, 5.73 | 0.38 | 1.42 | 0.42, 4.80 | 0.57 | 1.42 | 0.42, 4.81 | 0.57 |

**Model 1:** crude model; **Model 2:** Adjusted for age, gender, education level, marital status, drinking status, and smoking status; **Model 3:** Adjusted for age, gender, education level, marital status, drinking status, smoking status, dyslipidemia (current use of lipid-lowering medications, and/or TG>150 mg/dL, HDL-C<40 mg/dL, LDL-C>160mg/dL, or TC>240mg/dL), BUN, eGFR, DM, and obesity status.

**Abbreviation:** HR, hazard ratio, CI, confidence interval; BUN, blood urea nitrogen; eGFR, estimated glomerular filtration rate; DM, Diabetes mellitus; TG, triglycerides; HDL-C, high-density lipoprotein cholesterol; LDL-C, low-density lipoprotein cholesterol; TC, total cholesterol

**Table S6A** Misclassification-adjusted Cox regression for incident hypertension and CVD based on posterior-probability weighting

| **Characteristic** | **Hypertension** | | | | | **CVD** | | | | |
| --- | --- | --- | --- | --- | --- | --- | --- | --- | --- | --- |
|  | **N** | **Event N** | **HR** | **95% CI** | **p-value** | **N** | **Event N** | **HR** | **95% CI** | **p-value** |
| **Total sleep trajectories** | 5,603 | 1,123 |  |  |  | 5,603 | 522 |  |  |  |
| steady high sleeper | 3,784 | 726 (19%) | Reference | Reference |  | 3,784 | 319 (8.4%) | Reference | Reference |  |
| steady low sleeper | 1,124 | 242 (22%) | 1.12 | 0.97, 1.30 | 0.13 | 1,124 | 122 (11%) | 1.21 | 0.98, 1.50 | 0.08 |
| **inverted U-shape sleeper** | 349 | 85 (24%) | 1.35 | 1.07, 1.69 | 0.01 | 349 | 45 (13%) | 1.47 | 1.07, 2.02 | 0.02 |
| U-shape sleeper | 346 | 70 (20%) | 0.88 | 0.46, 1.68 | 0.69 | 346 | 36 (10%) | 0.79 | 0.32, 1.96 | 0.61 |
| **Nocturnal sleep trajectories** | 5,603 | 1,123 |  |  |  | 5,603 | 522 |  |  |  |
| steady high sleeper | 3,849 | 744 (19%) | Reference | Reference |  | 3,849 | 329 (8.5%) | Reference | Reference |  |
| steady low sleeper | 1,073 | 224 (21%) | 0.5 | 0.12, 2.04 | 0.33 | 1,073 | 117 (11%) | 0.91 | 0.49, 1.68 | 0.76 |
| inverted U-shape sleeper | 386 | 91 (24%) | 1.16 | 0.18, 7.68 | 0.88 | 386 | 45 (12%) | 0.50 | 0.16, 1.57 | 0.24 |
| U-shape sleeper | 295 | 64 (22%) | 1.46 | 0.45, 4.73 | 0.53 | 295 | 31 (11%) | 1.05 | 0.59, 1.86 | 0.88 |

**Note:** Latent classes were derived via latent class analysis using a three-step framework. Class assignment was based on the maximum posterior probability, and posterior-probability weighting was applied in Cox models to minimise bias due to misclassification. Covariates matched those used in the primary analysis.

**Table S6B** Misclassification-adjusted Cox regression for incident stroke and heart disease based on posterior-probability weighting

| **Characteristic** | **Stroke** | | | | | **Heart disease** | | | | |
| --- | --- | --- | --- | --- | --- | --- | --- | --- | --- | --- |
|  | **N** | **Event N** | **HR** | **95% CI** | **p-value** | **N** | **Event N** | **HR** | **95% CI** | **p-value** |
| **Total sleep trajectories** | 5,603 | 100 |  |  |  | 5,603 | 438 |  |  |  |
| steady high sleeper | 3,784 | 53 (1.4%) | Reference | Reference |  | 3,784 | 276 (7.3%) | Reference | Reference |  |
| steady low sleeper | 1,124 | 28 (2.5%) | 1.55 | 0.26, 9.32 | 0.63 | 1,124 | 99 (8.8%) | 1.14 | 0.90, 1.44 | 0.29 |
| inverted U-shape sleeper | 349 | 9 (2.6%) | 1.89 | 0.92, 3.88 | 0.08 | 349 | 36 (10%) | 1.34 | 0.94, 1.90 | 0.11 |
| U-shape sleeper | 346 | 10 (2.9%) | 0.86 | 0.13, 5.65 | 0.87 | 346 | 27 (7.8%) | 0.80 | 0.29, 2.25 | 0.68 |
| **Nocturnal sleep trajectories** | 5,603 | 100 |  |  |  | 5,603 | 438 |  |  |  |
| steady high sleeper | 3,849 | 52 (1.4%) | Reference | Reference |  | 3,849 | 290 (7.5%) | Reference | Reference |  |
| steady low sleeper | 1,073 | 29 (2.7%) | 0.57 | 0.14, 2.34 | 0.43 | 1,073 | 91 (8.5%) | 1.09 | 0.76, 1.57 | 0.63 |
| **inverted U-shape sleeper** | 386 | 11 (2.8%) | 1.69 | 1.06, 2.71 | 0.03 | 1.69 | 34 (8.8%) | 1.08 | 0.85, 1.38 | 0.54 |
| U-shape sleeper | 295 | 8 (2.7%) | 1.63 | 0.48, 5.53 | 0.44 | 295 | 23 (7.8%) | 1.46 | 0.44, 4.84 | 0.54 |

**Note:** Latent classes were derived via latent class analysis using a three-step framework. Class assignment was based on the maximum posterior probability, and posterior-probability weighting was applied in Cox models to minimise bias due to misclassification. Covariates matched those used in the primary analysis.

**Table S7A** Association of the total/nocturnal sleep trajectories and new-onset hypertension/CVD after additional adjustment for socioeconomic status

| **Characteristic** | **Hypertension** | | | | | **CVD** | | | | |
| --- | --- | --- | --- | --- | --- | --- | --- | --- | --- | --- |
|  | **N** | **Event N** | **HR** | **95% CI** | **p-value** | **N** | **Event N** | **HR** | **95% CI** | **p-value** |
| **Socioeconomic status≤4** |  |  |  |  |  |  |  |  |  |  |
| **Total sleep trajectories** | 3,104 | 647 |  |  |  | 3,104 | 293 |  |  |  |
| steady high sleeper | 2,066 | 411 (20%) | Reference | Reference |  | 2,066 | 178 (8.6%) | Reference | Reference |  |
| steady low sleeper | 621 | 135 (22%) | 1.11 | 0.91, 1.35 | 0.32 | 621 | 65 (10%) | 1.16 | 0.87, 1.55 | 0.30 |
| **inverted U-shape sleeper** | 204 | 54 (26%) | 1.45 | 1.09, 1.93 | 0.01 | 204 | 30 (15%) | 1.58 | 1.06, 2.34 | 0.03 |
| U-shape sleeper | 213 | 47 (22%) | 1.00 | 0.73, 1.36 | ≥0.99 | 213 | 20 (9.4%) | 0.95 | 0.59, 1.52 | 0.83 |
| **Nocturnal sleep trajectories** | 3,104 | 647 |  |  |  | 3,104 | 293 |  |  |  |
| steady high sleeper | 2,091 | 412 (20%) | Reference | Reference |  | 2,091 | 179 (8.6%) | Reference | Reference |  |
| steady low sleeper | 600 | 137 (23%) | 1.15 | 0.94, 1.40 | 0.18 | 600 | 68 (11%) | 1.23 | 0.92, 1.65 | 0.16 |
| inverted U-shape sleeper | 231 | 55 (24%) | 1.26 | 0.95, 1.68 | 0.11 | 231 | 29 (13%) | 1.38 | 0.92, 2.05 | 0.12 |
| U-shape sleeper | 182 | 43 (24%) | 1.65 | 0.66, 4.14 | 0.29 | 182 | 17 (9.3%) | 0.97 | 0.24, 3.95 | 0.96 |
| **Socioeconomic status>4** |  |  |  |  |  |  |  |  |  |  |
| **Total sleep trajectories** | 2,499 | 476 |  |  |  | 2,499 | 229 |  |  |  |
| steady high sleeper | 1,718 | 315 (18%) | Reference | Reference |  | 1,718 | 141 (8.2%) | Reference | Reference |  |
| steady low sleeper | 145 | 31 (21%) | 1.21 | 0.83, 1.75 | 0.32 | 145 | 15 (10%) | 1.24 | 0.73, 2.13 | 0.43 |
| inverted U-shape sleeper | 503 | 107 (21%) | 1.16 | 0.93, 1.45 | 0.19 | 503 | 57 (11%) | 1.28 | 0.94, 1.75 | 0.12 |
| U-shape sleeper | 133 | 23 (17%) | 0.95 | 0.62, 1.45 | 0.80 | 133 | 16 (12%) | 1.38 | 0.82, 2.33 | 0.22 |
| **Nocturnal sleep trajectories** | 2,499 | 476 |  |  |  | 2,499 | 229 |  |  |  |
| steady high sleeper | 1,758 | 332 (19%) | Reference | Reference |  | 1,758 | 150 (8.5%) | Reference | Reference |  |
| steady low sleeper | 473 | 87 (18%) | 0.98 | 0.77, 1.26 | 0.89 | 473 | 49 (10%) | 1.19 | 0.85, 1.65 | 0.32 |
| inverted U-shape sleeper | 155 | 36 (23%) | 1.24 | 0.87, 1.75 | 0.23 | 155 | 16 (10%) | 1.18 | 0.70, 1.98 | 0.54 |
| U-shape sleeper | 113 | 21 (19%) | 1.44 | 0.41, 5.06 | 0.57 | 113 | 14 (12%) | 2.48 | 0.50, 12.31 | 0.27 |

**Model 3:** Adjusted for age, gender, marital status, drinking status, smoking status, dyslipidemia (current use of lipid-lowering medications, and/or TG>150 mg/dL, HDL-C<40 mg/dL, LDL-C>160mg/dL, or TC>240mg/dL), BUN, eGFR, DM, and obesity status.

**Table S7B** Association of the total/nocturnal sleep trajectories and new-onset stroke/heart disease after additional adjustment for socioeconomic status

| **Characteristic** | **Stroke** | | | | | **Heart disease** | | | | |
| --- | --- | --- | --- | --- | --- | --- | --- | --- | --- | --- |
|  | **N** | **Event N** | **HR** | **95% CI** | **p-value** | **N** | **Event N** | **HR** | **95% CI** | **p-value** |
| **Socioeconomic status≤4** |  |  |  |  |  |  |  |  |  |  |
| **Total sleep trajectories** | 3,104 | 51 |  |  |  | 3,104 | 248 |  |  |  |
| steady high sleeper | 2,066 | 25 (1.2%) | Reference | Reference |  | 2,066 | 157 (7.6%) | Reference | Reference |  |
| steady low sleeper | 621 | 17 (2.7%) | 2.18 | 1.16, 4.10 | 0.02 | 621 | 50 (8.1%) | 1.01 | 0.73, 1.39 | 0.97 |
| inverted U-shape sleeper | 204 | 5 (2.5%) | 1.87 | 0.70, 4.99 | 0.21 | 204 | 25 (12%) | 1.48 | 0.97, 2.28 | 0.07 |
| U-shape sleeper | 213 | 4 (1.9%) | 1.16 | 0.38, 3.53 | 0.79 | 213 | 16 (7.5%) | 0.87 | 0.51, 1.46 | 0.59 |
| **Nocturnal sleep trajectories** | 3,104 | 51 |  |  |  | 3,104 | 248 |  |  |  |
| steady high sleeper | 2,091 | 17 (2.7%) | Reference | Reference |  | 2,091 | 50 (8.1%) | Reference | Reference |  |
| steady low sleeper | 600 | 25 (1.2%) | 1.73 | 0.90, 3.32 | ≥0.99 | 600 | 157 (7.6%) | 1.1 | 0.80, 1.52 | 0.55 |
| inverted U-shape sleeper | 231 | 5 (2.5%) | 3.07 | 1.24, 7.58 | 0.02 | 231 | 25 (12%) | 1.29 | 0.83, 2.00 | 0.25 |
| U-shape sleeper | 182 | 4 (1.9%) | 0.28 | 0.01, 7.37 | 0.45 | 182 | 16 (7.5%) | 1.19 | 0.25, 5.67 | 0.82 |
| **Socioeconomic status>4** |  |  |  |  |  |  |  |  |  |  |
| **Total sleep trajectories** | 2,499 | 49 |  |  |  | 2,499 | 190 |  |  |  |
| steady high sleeper | 1,718 | 28 (1.6%) | Reference | Reference |  | 1,718 | 119 (6.9%) | Reference | Reference |  |
| steady low sleeper | 145 | 4 (2.8%) | 1.67 | 0.58, 4.79 | 0.34 | 145 | 11 (7.6%) | 1.08 | 0.58, 2.02 | 0.81 |
| inverted U-shape sleeper | 503 | 11 (2.2%) | 1.21 | 0.59, 2.46 | 0.60 | 503 | 49 (9.7%) | 1.30 | 0.93, 1.83 | 0.13 |
| U-shape sleeper | 133 | 6 (4.5%) | 3.05 | 1.25, 7.44 | 0.01 | 133 | 11 (8.3%) | 1.09 | 0.58, 2.03 | 0.79 |
| **Nocturnal sleep trajectories** | 2,499 | 49 |  |  |  | 2,499 | 190 |  |  |  |
| steady high sleeper | 1,758 | 25 (1.4%) | Reference | Reference |  | 1,758 | 133 (7.6%) | Reference | Reference |  |
| steady low sleeper | 473 | 13 (2.7%) | 1.93 | 0.96, 3.86 | 0.06 | 473 | 38 (8.0%) | 1.04 | 0.71, 1.50 | 0.85 |
| inverted U-shape sleeper | 155 | 6 (3.9%) | 1.51 | 0.57, 3.98 | 0.41 | 155 | 10 (6.5%) | 0.78 | 0.41, 1.50 | 0.46 |
| U-shape sleeper | 113 | 5 (4.4%) | 3.65 | 0.15, 87.44 | 0.42 | 113 | 9 (8.0%) | 2.1 | 0.32, 13.90 | 0.44 |

**Model 3:** Adjusted for age, gender, marital status, socioeconomic status, drinking status, smoking status, dyslipidemia (current use of lipid-lowering medications, and/or TG>150 mg/dL, HDL-C<40 mg/dL, LDL-C>160mg/dL, or TC>240mg/dL), BUN, eGFR, DM, and obesity status.
