# Supplementary material for: Subsequent Hypertension’s Mediation of the Association Between Sleep Duration Trajectories and New-Onset Cardiovascular Disease: Population-Based Cohort Study
Source: JMIR Aging. 2026 May 28;9:e78914. doi: 10.2196/78914 (PMC13218652; doi:10.2196/78914)
Supplement: Multimedia Appendix 2 [file aging-v9-e78914-s002.docx]

**Subsequent hypertension mediates the association between sleep duration trajectories and new-onset cardiovascular disease: a population-based cohort study**


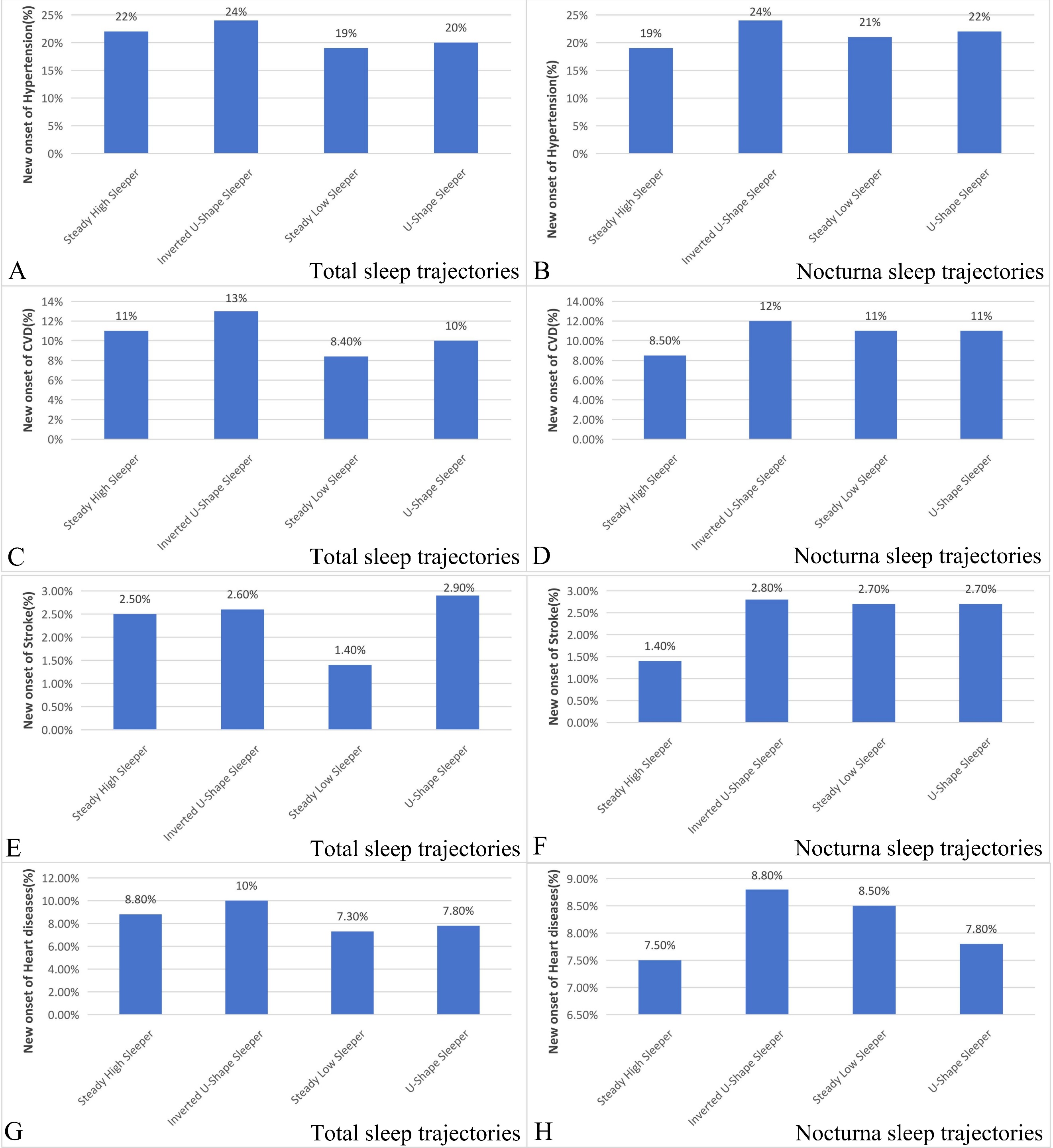


**Supplementary Figure 1** Total sleep/nocturnal sleep and new onset rates of hypertension (**A, B**), cardiovascular diseases (**C, D**), stroke (**E, F**), and heart diseases (**G, H**) according to total/nocturnal sleep duration trajectories

**Abbreviation:** CVD, cardiovascular disease


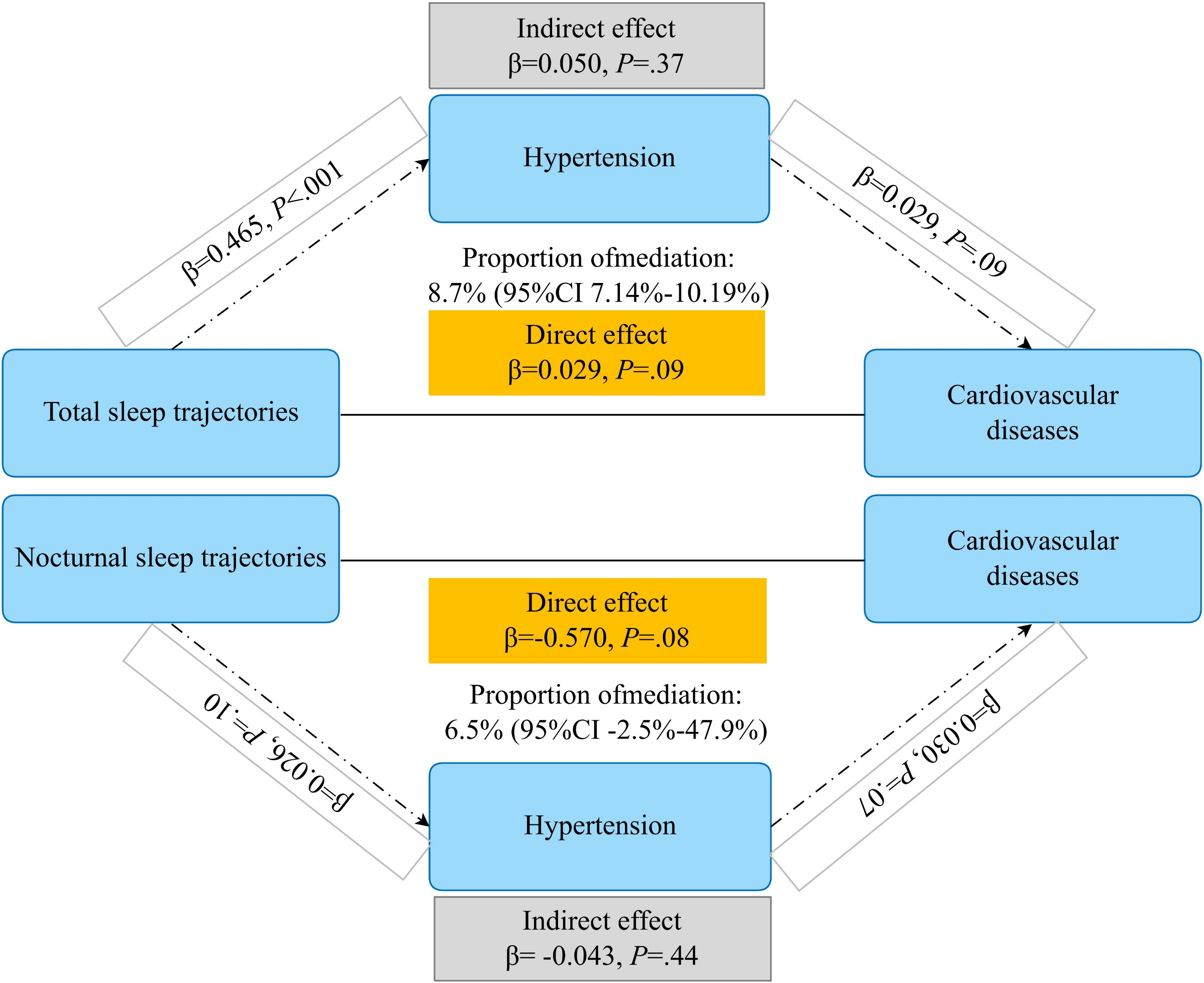


**Supplementary Figure 2** Mediation analysis of the association between the total sleep/nocturnal sleep duration trajectories and CVD

**Abbreviation:** CVD, cardiovascular disease; HR, hazard ratio, CI, confidence interval;


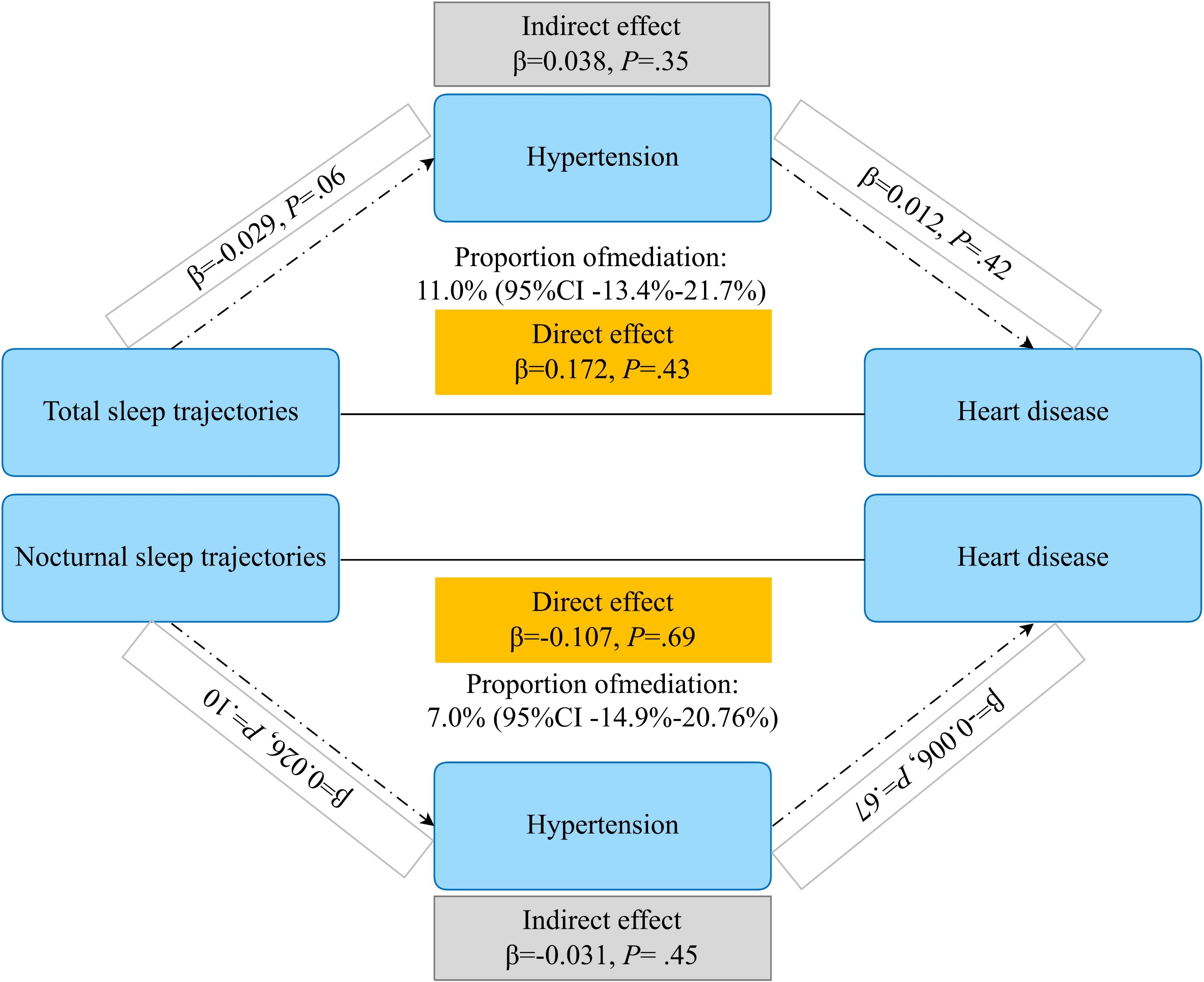


**Supplementary Figure 3** Mediation analysis of the association between the total sleep/nocturnal sleep duration trajectories heart diseases

**Abbreviation:** HR, hazard ratio, CI, confidence interval;
